# Comparative Genomic Analysis Reveals Extensive Genetic Variations of WRKYs in Solanaceae and Functional Variations of CaWRKYs in Pepper

**DOI:** 10.3389/fgene.2019.00492

**Published:** 2019-05-28

**Authors:** Yuan Cheng, Golam Jalal Ahammed, Zhuping Yao, Qingjing Ye, Meiying Ruan, Rongqing Wang, Zhimiao Li, Guozhi Zhou, Hongjian Wan

**Affiliations:** ^1^State Key Laboratory Breeding Base for Zhejiang Sustainable Pest and Disease Control, Institute of Vegetables, Zhejiang Academy of Agricultural Sciences, Hangzhou, China; ^2^College of Forestry, Henan University of Science and Technology, Luoyang, China

**Keywords:** WRKY, solanaceae, genetic variation, genetic conservatism, functional diversity

## Abstract

As a conserved protein family, WRKY has been shown to be involved in multiple biological processes in plants. However, the mechanism of functional diversity for WRKYs in pepper has not been well elucidated. Here, a total of 223 WRKY members from solanaceae crops including pepper, tomato and potato, were analyzed using comparative genomics. A tremendous genetic variation among WRKY members of different solanaceous plants or groups was demonstrated by the comparison of some WRKY features, including number/size, group constitution, gene structure, and domain composition. The phylogenetic analysis showed that except for the known WRKY groups (I, IIa/b/c/d/e and III), two extra WRKY subgroups specifically existed in solanaceous plants, which were named group IIf and group IIg in this study, and their genetic variations were also revealed by the characteristics of some group IIf and IIg WRKYs. Except for the extensive genetic variations, certain degrees of conservatism for solanaceae WRKYs were also revealed. Moreover, the variant zinc-finger structure (CX_4,7_CX_22-24_HXC) in group III of solanaceae WRKYs was identified. Expression profiles of *CaWRKY* genes suggested their potential roles in pepper development and stress responses, and demonstrated a functional division pattern for pepper CaWRKYs. Furthermore, functional analysis using virus induced gene silencing (VIGS) revealed critical roles of two CaWRKYs (CaWRKY45 and CaWRKY58) in plant responses to disease and drought, respectively. This study provides a solid foundation for further dissection of the evolutionary and functional diversity of solanaceae WRKYs in crop plants.

## Introduction

The WRKY transcription factor (TF) family is one of the largest TF families in higher plants, which has been extensively studied for its important regulatory roles in multiple biological processes relating to plant growth, development and responses to stress (Pandey and Somssich, 2009; Li et al., 2013a; Dang et al., 2014; Cai et al., 2015). As a sequence specific DNA-binding TFs, the WRKYproteins contain at least one WRKY domain, which is responsible for the binding of special *cis*-element (w-box: TTGACC/T) (Rushton et al., 2010). As the common features of WRKY TFs, the WRKY domain is typically composed of a highly conserved peptide (WRKYGQK motif) and a zinc-finger structure (C_2_H_2_:CX_4-5_CX_22-23_HXH or C_2_HC:CX_7_CX_23_HXC) (Rushton et al., 1996, 2010; Eulgem and Somssich, 2007), which form a four-stranded β-sheet and a zinc-binding pocket formed by the conserved Cys/His residues located at one end of the β-sheet (Eulgem et al., 2000; Xie et al., 2005; Yamasaki et al., 2005).

Based on the number of WRKY domains and the corresponding zinc-finger structure, the WRKY proteins were initially classified into three groups, namely group I, II, and III (Eulgem et al., 2000). The group I WRKY members contain two WRKY domains with two CX_4_CX_22-23_HXH zinc-finger motifs, the group II WRKYs have one WRKY domain with one CX_4_CX_22-23_HXH zinc-finger motif, and the group III WRKYs contain one WRKY domain with one CX_7_CX_23_HXC zinc-finger motif (Rushton et al., 1996, 2010; Eulgem and Somssich, 2007). The group II WRKYs could be further divided into five subgroups, such as subgroup IIa, IIb, IIc, IId, and IIe (Eulgem et al., 2000; Xie et al., 2005). However, later studies on phylogenetic relationship reclassified the group II into IIa+IIb, II c, and IId+IIe (Wu et al., 2005; Zhang and Wang, 2005; Huang et al., 2012). It is well recognized that WRKY proteins originated from the lower photosynthetic and non-photosynthetic eukaryotes. As the group I WRKY members only exist in lower plants, they are now believed to be the evolutionary ancestors of the other WRKYs (Wu et al., 2005; Zhang and Wang, 2005; Cheng et al., 2012).

Since the first identification of plant WRKY protein in sweet potato (Ishiguro and Nakamura, 1994), the whole genome level WRKY identification has been accomplished in a wide variety of plant species (Wu et al., 2005; Zhang and Wang, 2005; Mangelsen et al., 2008; Rushton et al., 2008; Ling et al., 2011; Tripathi et al., 2012), including Arabidopsis (Rushton et al., 2010), rice (Ross et al., 2007), tomato (Huang et al., 2012) and potato (Huang and Liu, 2013). Many identified WRKYs have been shown to be functional in a broad range of biological processes, including biotic or abiotic stress responses (Li et al., 2013b; Dang et al., 2014; Zhou et al., 2015), hormone responses (Antoni et al., 2011; Bakshi and Oelmuller, 2014; Zhang et al., 2015) and other developmental processes (Robatzek and Somssich, 2002; Miao et al., 2004; Jiang and Yu, 2009; Zentgraf et al., 2010; Suttipanta et al., 2011; Besseau et al., 2012). SlWRKY39, a group IIa member in tomato, was reported to play important role in disease resistance against *Pseudomonas syringae* pv. tomato DC3000 (*Pst*DC3000), as well as in osmotic stress and drought tolerance (Sun et al., 2015). Another group I member defined as SlDRW1, is required for disease resistance against *Botrytis cinerea* and tolerance to oxidative stress in tomato (Liu et al., 2014). In pepper, CaWRKY40 from group IIa, plays an important role in the modulation of both high temperature tolerance and *Ralstonia solanacearum* resistance, and this regulation seems to be dependent on the transcriptional activity of CaWRKY06 (group IIb member) (Dang et al., 2013; Cai et al., 2015). In potato, the function of WRKY TF was barely reported, except for the StWRKY01 (group II member), which might play certain roles in the defense against *Erwinia carotovora* subsp. *atroseptica* and *Phytophthora infestans* (Dellagi et al., 2000). Despite being a highly conserved protein family characterized by the conserved WRKY-domain and specific w-box (TTGACC/T) recognition pattern, the functional diversity of WRKY TFs has not been systematically elucidated yet. So far, several hypotheses, including the specificity determinant of the nucleotides sequence adjacent to the W-box core sequence (Ciolkowski et al., 2008), the temporal and spatial expression differences of *WRKY* genes decided by their own structural variations, and interacting partners (co-activators, chromatin remodelers) modulating patterns (Andreasson et al., 2005; Qiu et al., 2008; Wang et al., 2010; Cheng et al., 2012), were proposed.

Although many WRKYs have been analyzed using bioinformatics tools in some solanaceous species, the genetic or functional diversity of solanaceae WRKYs has barely been studied. Thus, based on the genome databases of the three representative solanaceous plants (pepper, potato and tomato) (The Potato Genome Sequencing Consortium, 2011; The Tomato Genome Consortium, 2012; Cheng et al., 2016), totally 223 solanaceae WRKY members were collected (Huang et al., 2012; Huang and Liu, 2013; Cheng et al., 2016), and their extensive genetic variations were comparatively analyzed in the current study. Moreover, the functional diversity of CaWRKYs in pepper was studied through the analysis of tissue-specific expression pattern and stress responses.

## Materials and Methods

### Collection of WRKY Proteins From Solanaceous Plant Genomes

The bioinformation of WRKYs (accession ID, group classification etc.) in pepper, tomato and potato were collected from previous publications (Huang et al., 2012; Huang and Liu, 2013; Cheng et al., 2016). The detail information of the WRKYs (gene/protein sequences) was further collected from Pepper Informatics Hub (PIH^1^), Sol Genomics Network (SGN^2^) and Potato Genome Project (PGP^3^) according to their corresponding accession IDs, respectively (Supplementary Table S1). The HMM profile of the conserved WRKY domain (Pfam: PF03106) from Pfam 27.0 database^4^ was used for the validation of all collected WRKY sequences.

### Phylogenetic Analysis and Intron-Exon Configuration of Solanaceae WRKY Genes

The alignments of amino acid sequences of 72 complete WRKY domains from pepper, 94 complete WRKY domains from tomato, 92 complete WRKY domains from potato, 84 complete WRKY domains from Arabidopsis and 102 complete WRKY domains from rice (both N- and C-terminal domains included), were performed using ClustalX 1.83 with default settings (Thompson et al., 1997)^5^. An unrooted phylogenetic tree was conducted based on the alignment data using MEGA 5.0 with neighbor joining method and maximum-likelihood method, respectively (Tamura et al., 2011). Relative branch support was evaluated using bootstraps (1000 replicates), branch lengths were calculated by pairwise comparison of genetic distances, and missing data were treated by pairwise deletions of gaps. The intron-exon structure was visualized using GSDS 2.0^6^ based on genomic DNA sequences of the 223 solanaceae (pepper, tomato and potato) WRKYs. The coefficient of variation (CV) on intron numbers was calculated by the formula of STDEV/AVERAGE in Excel 2007 (Supplementary Table S1).

### WRKY Domain Structure and Motif Analyses

The specific WRKY domain sequences of all WRKYs from pepper, tomato and potato were collected using SMART^7^. The WRKY domain sequences were aligned using ClustalX 1.83 with default settings (see Footnote 5), converted to “CLUSTAL” format and visualized by Bioedit 7.0 (Supplementary Table S2). The sequence logos of WRKY domains were generated online using weblogo^8^. All retrieved WRKY sequences were subjected to domain analysis by using the Conserved Domain Database (CDD) programs^9^ (Supplementary Table S3).

### Chromosome Mapping and Comparative Analysis of WRKYs Among Solanaceous Plants

Chromosome mapping of 223 solanaceae *WRKY* genes was performed with MapDraw V2.1 (Zheng et al., 2006) based on their gene chromosomal localization information derived from SGN, PGR and PGD, respectively. Online platform PGDD^10^ was used for comparative genomics analysis of *WRKY*s from pepper, tomato and potato. Based on the constructed chromosome mappings, correlative tomato-potato-pepper *WRKY* genes were labeled and connected with black lines.

### Expression Analysis of *CaWRKY* Genes and Virus-Induced Gene Silencing (VIGS) of *CaWRKY*s

Total RNA was isolated from collected pepper samples (Root, stem, Leaf, flower, bud, green fruit, red fruit, and seed). Total RNA kit according to the manufacturer’s protocol (Tiangen, Beijing, China), and reverse transcribed into cDNA using the FastQuant RT Kit (Tiangen, Beijing, China). Genscript online tool ^11^ was used for the total 61 pairs of *CaWRKY*-specific primers design (Supplementary Table S4). The real-time PCR reactions were carried out in 20 μl reaction mixture containing 10 μl SuperMix, 0.4 μl of each primer (20 μM), 1 μl diluted (10×) sample cDNA, and 8.2 μl sterile distilled water. RT-qPCR assay was performed using the following program: 30 s at 94°C, followed by 40 cycles of 5 s at 94°C, 15 s at 55°C and 10 s at 72°C. The pepper gene *UBI-1* (Capana04g000407) (F: AAGGAAATGTGTGTCTCAAC; R: TCCAAATGCCAAACTTCTAG) was used as an internal control for the normalization of expression levels of the target genes (Livak and Schmittgen, 2001). The relative gene expression was calculated according to Livak and Schmittgen (2001). Three independent biological replicates were performed.

Due to highly conservation among pepper CaWRKY members, SGN VIGS online tool^12^ was used to ensure the specificity of target *CaWRKY* genes. The virus-induced gene silencing (VIGS) target fragments of *CaWRKY22*, *CaWRKY45*, and *CaWRKY58* were amplified with gene-specific primers (Supplementary Table S5) and ligated into the VIGS vector pTRV, which was subsequently transformed into *Agrobacterium tumefaciens* strain GV3101 for VIGS analyses. VIGS analyses were performed as described previously (Wang et al., 2015). The empty pTRV vector was used as control. Four weeks after agro-infiltration, pepper plants were infected with *Pst*DC3000 and *B. cinerea*. The bacterial strain *Pst*DC3000 were grown overnight in King’s B medium containing rifampicin (50 mg/mL) and kanamycin (25 mg/mL). The bacterial cells were harvested and suspended in 10 mM MgCl_2_. The cells were then diluted to 1 × 10^6^ cfu/ml and infiltrated into the abaxial surface of the leaves (Cheng et al., 2012). Growth of *B. cinerea* were performed as previously described (Zheng et al., 2006), and the inoculation was conducted by spot infection of 2 μl bacterial fluids (2 × 10^5^ spor/ml). For abiotic stress treatments, 4-week-old pepper seedlings were exposed to osmotic stress by irrigation of 20% PEG6000 for 12 h, or exposed to drought by withholding water for 9 d, or to heat by elevating the temperature to 42°C for 3 h, and leaves were collected at the end of each treatment. The relative water content (%) in leaves was measured following the drying method (Wang et al., 2018). All materials were frozen in liquid nitrogen and used for the following expression analyses. For each treatment, three replicates (four plants in one replicate) were examined. DMRT (Duncan’s multiple range test) was used for statistical analysis.

## Results

### Comprehensive Evolutionary Analysis of WRKY Proteins in Solanaceous Plants

Here, totally 223 solanaceae WRKYs, as well as the WRKYs of two model plants (100 OsWRKYs from rice, 75 AtWRKYs from Arabidopsis), were collected for comparative analysis together. According to Table 1, the number of WRKY proteins is not linearly correlated with the genome size of the corresponding plant species (Table 1). For example, with the very large genome size of 3500-magabase, only 61 CaWRKYs were detected from pepper. On the contrary, totally 100 WRKYs were identified from rice (only 390-magabase genome) (Table 1).

**Table 1:** Group constitution of WRKYs in Solanaceae (pepper, tomato and potato) and other plants (rice and Arabidopsis).

WRKY group		Solanaceae(900M)	Proportion %	Pepper(3500M)	Proportion %	Tomato(900M)	Proportion %	Potato(850M)	Proportion %	Rice(390M)	Proportion %	Arabidopsis (125M)	Proportion %
Group I		41	18.4	13	21.3	15	18.5	13	16.0	12	12.0	15	20.0
Group II		137	61.4	35	57.3	52	64.3	50	61.7	42	42.0	41	54.7
	II a	14	6.3	4	6.6	5	6.2	5	6.2	3	3.0	3	4.0
	II b	20	9.0	6	9.8	8	9.9	6	7.4	8	8.0	8	10.7
	II c	44	19.7	12	19.7	16	19.8	16	19.8	15	15.0	15	20.0
	II d	18	8.1	5	8.2	6	7.4	7	8.6	6	6.0	7	9.3
	II e	21	9.4	6	9.8	8	9.9	7	8.6	10	10.0	8	10.7
	IIf	5	2.2	1	1.6	1	1.2	3	3.7	0	0.0	0	0.0
	IIg	15	6.7	1	1.6	8	9.9	6	7.4	0	0.0	0	0.0
Group III		34	15.2	9	14.8	11	13.6	14	17.3	31	31.0	14	18.7
NG		11	4.9	4	6.6	3	3.7	4	4.9	15	15.0	5	6.7
Total		223	100.0	61	100.0	81	100.0	81	100.0	100	100.0	75	100.0


Although pepper, tomato and potato all belong to the solanaceae family, they could be further differentiated into genus levels, such as the *Capsicum* genus (pepper) and *Solanum* genus (tomato, potato), respectively. According to Table 1, some genetic variations on WRKY numbers were reflected on genus level, as the number of WRKYs in tomato and potato (the *Solanum* genus) are both 81, but only 61 WRKY members in pepper (the *Capsicum* genus) were identified, indicating the occurrence of *WRKY* gene loss/gain events during the evolution of solanaceous plants. Further analysis in group-wise manner showed that the events of gene loss/gain were mainly occurred in group II (especially IIc and IIg), as there are 35 group II CaWRKYs in pepper (including 2 IIc, 1 IIg), 52 group II SlWRKYs in tomato (including 16 IIc, 8 IIg), and 50 group II StWRKYs in potato (including 16 IIc, 6 IIg), respectively (Table 1).

As shown in Table 2, the average size [encoding nucleotide residues (bp) and amino acids (aa)] of CaWRKYs, SlWRKYs, and StWRKYs are 1075.0bp/355.7aa, 1060.0bp/352.3aa, and 1017.4bp/388.1aa, respectively, suggesting the presence of size variations in WRKY families of different solanaceous plants. Moreover, the distinctive differences on the average WRKY size of most groups between pepper (the *Capsicum* genus) and tomato/potato (the *Solanum* genus) were observed (Table 2). The average size of different WRKY groups were obviously more similar between tomato and potato, the average size of pepper WRKY groups were either larger (group I, IIa, IIf, and IIg) or smaller (for IIb, IIc, IId, IIe, and III) than their counterparts in tomato and potato.

**Table 2:** Group classification and length of encoding nucleotide residues (bp)/amino acids (aa) of WRKYs in pepper, tomato and potato.

WRKY group		WRKY Name	Length of encoding nucleotide residues (bp) and amino acids (aa)	Average length			
				
				Solanaceae (bp/aa)	Pepper (bp/aa)	Tomato (bp/aa)	Potato (bp/aa)
Group I		CaWRKY13;CaWRKY21;CaWRKY24;CaWRKY25; CaWRKY28;CaWRKY31;CaWRKY33;CaWRKY37;CaWRKY38; CaWRKY45;CaWRKY47;CaWRKY51;CaWRKY53; SlWRKY01;SlWRKY02;SlWRKY03; SlWRKY04;SlWRKY05;SlWRKY14; SlWRKY15;SlWRKY18;SlWRKY20;SlWRKY31;SlWRKY32; SlWRKY33;SlWRKY34;SlWRKY36;SlWRKY44;StWRKY01; StWRKY02;StWRKY03;StWRKY04;StWRKY05; StWRKY38;StWRKY39;StWRKY40;StWRKY41;StWRKY42; StWRKY43;StWRKY44;StWRKY58	1305/434;1851/616;1464/487;1524/507;1647/548;1368/455;2610/869;1764/587;2232/743;1641/546;2112/703;1260/419;1473/490;1242/413;2220/739;1383/460;1527/508;1461/486;1248/415;2112/703;1791/596;1836/611;1608/535;1542/513;1590/529;1380/459;1206/401;1269/422;2247/748;2094/697;1527/508;1551/516;1356/451;1605/534;1227/408;1836/611;1791/596;1374/457;1650/549;1269/422;1404/467	1624.3/540.4	1711.6/569.5	1561/519.3	1610.1/535.7
Group II	IIa	CaWRKY15;CaWRKY27;CaWRKY40;CaWRKY55; SlWRKY39;SlWRKY40; SlWRKY43;SlWRKY45;SlWRKY46;StWRKY48; StWRKY49;StWRKY50;StWRKY51;StWRKY52	1083/360;1086/361;795/264;729/242;1053/350;1083/360;651/216;777/258;762/253;1083/360;1068/355;786/261;762/253;654/217	883.7/293.6	923.3/306.8	865.2/287.4	870.6/289.2
	IIb	CaWRKY09;CaWRKY11;CaWRKY16;CaWRKY26;CaWRKY34; CaWRKY43;SlWRKY06; SlWRKY09;SlWRKY16;SlWRKY17;SlWRKY72; SlWRKY73;SlWRKY74; SlWRKY76;StWRKY06; StWRKY07;StWRKY08;StWRKY12; StWRKY78;StWRKY79	1368/455;1584/527;975/324;1896/631;1764/587;1497/498;1653/550;1428/475;1497/498;1896/631;1323/440;1575/524;1950/649;1179/392;1662/553;1806/601;1491/496;1242/413;1332/443;1584/527	1535.1/510.7	1514/503.7	1562.6/519.9	1519.5/505.5
	IIc	CaWRKY03;CaWRKY04;CaWRKY14;CaWRKY36; CaWRKY39;CaWRKY44;CaWRKY54;CaWRKY56; CaWRKY57; CaWRKY58;CaWRKY59;CaWRKY61;SlWRKY12; SlWRKY13;SlWRKY23;SlWRKY28;SlWRKY30; SlWRKY38;SlWRKY47; SlWRKY48;SlWRKY50;SlWRKY51;SlWRKY55;SlWRKY56; SlWRKY57;SlWRKY61;SlWRKY71;SlWRKY75; StWRKY15; StWRKY16;StWRKY23;StWRKY24;StWRKY33; StWRKY34;StWRKY35;StWRKY59;StWRKY60;StWRKY61; StWRKY62;StWRKY63;StWRKY68; StWRKY69;StWRKY80;StWRKY81	684/227;720/239;483/160;957/318;501/166;663/220;924/307;564/187;702/233;414/137;513/170;672/223;711/236;705/234;963/320;1008/335;969/322;396/131;975/324;888/295;546/181;525/174;687/228;696/231;984/327;567/188;954/317;519/172;756/251;705/234;960/317;753/250;957/318;960/319;1005/334;1020/339;696/231;753/250;498/165;309/102;687/228;981/326;519/172;426/141	724.4/240.4	649.8/215.6	755.8/250.9	749.1/248.6
	IId	CaWRKY08;CaWRKY22;CaWRKY23; CaWRKY30;CaWRKY60;SlWRKY07; SlWRKY08;SlWRKY10;SlWRKY11;SlWRKY21;SlWRKY24; StWRKY09;StWRKY10; StWRKY11;StWRKY13;StWRKY14;StWRKY18;StWRKY19	558/185;1020/339;1053/350;801/266;993/330;1056/351;981/326;1017/338;987/328;1047/348;996/331;1065/354;975/324;1005/334;1005/334;939/312;1041/346;1068/355	978.2/325.1	885/294	1014/337	1014/337
	IIe	CaWRKY02;CaWRKY07;CaWRKY10;CaWRKY35; CaWRKY41;CaWRKY48; SlWRKY22;SlWRKY25; SlWRKY29;SlWRKY35;SlWRKY37;SlWRKY77;SlWRKY78; SlWRKY79;StWRKY20;StWRKY36; StWRKY46;StWRKY47;StWRKY70;StWRKY71; StWRKY72;	972/323;1089/362;912/303;804/267;1122/373;1149/382;1068/355;936/311;912/303;1146/381;723/240;768/255;828/275;927/308;1059/352;933/310;1242/413;735/244;900/299;771/256;627/208	934.4/310.5	1008/335	913.5/303.5	895.3/297.4
	IIf	CaWRKY17;SlWRKY26;StWRKY17;StWRKY22;StWRKY73	786/261;660/219;780/259;702/233;522/173	690/229	786/261	660/219	668/221.7
	IIg	CaWRKY19;SlWRKY62;SlWRKY63;SlWRKY64; SlWRKY65;SlWRKY66;SlWRKY67; SlWRKY68;SlWRKY69;StWRKY25; StWRKY26;StWRKY27;StWRKY28;StWRKY29;StWRKY30	1011/336;837/278;969/322;969/322;717/238;966/321;762/253;1080/359;1101/366;993/330;993/330;750/249;936/311;750/249;996/331	922/306.3	1011/336	925.1/307.4	903/300
Group III		CaWRKY05;CaWRKY06;CaWRKY18;CaWRKY20;CaWRKY29; CaWRKY32;CaWRKY42;CaWRKY49;CaWRKY50; SlWRKY19;SlWRKY41;SlWRKY42;SlWRKY52;SlWRKY53; SlWRKY54; SlWRKY58;SlWRKY59;SlWRKY60; SlWRKY80;SlWRKY81;StWRKY37;StWRKY53;StWRKY54; StWRKY55;StWRKY56;StWRKY57;StWRKY64;StWRKY65; StWRKY66;StWRKY67;StWRKY74;StWRKY75;StWRKY76; StWRKY77;	744/247;762/253;1098/365;900/299;684/227;594/197;990/329;648/215;699/232;732/243;1011/336;873/29;1062/353;1083/360;1020/339;984/327;735/244;588/195;822/273;876/291;873/290;1071/356;1041/346;891/294;906/301;996/331;1083/360;1020/339;918/305;1044/347;834/277;882/293;657/218;672/223	876.3/291.0	791/262.7	889.6/295.5	920.6/305.7
NG		CaWRKY01;CaWRKY12;CaWRKY46;CaWRKY52; SlWRKY27;SlWRKY49;SlWRKY70;StWRKY21; StWRKY31;StWRKY32;StWRKY45	573/190;912/303;861/286;1017/338;537/178;873/290;873/290;717/238;645/214;501/166;486/161	726.8/232.2	840.8/254.3	761.3/252.7	587.3/194.8


It is noteworthy that although both tomato and potato belong to the same genus, as well as owning the same number of WRKY members, some unconspicuous variations on WRKY group features, including WRKY group constitution and average size of group members, were also detected (Tables 1, 2).

### Phylogenetic Analysis of WRKY Gene Family in Solanaceous Plants

**FIGURE 1:**
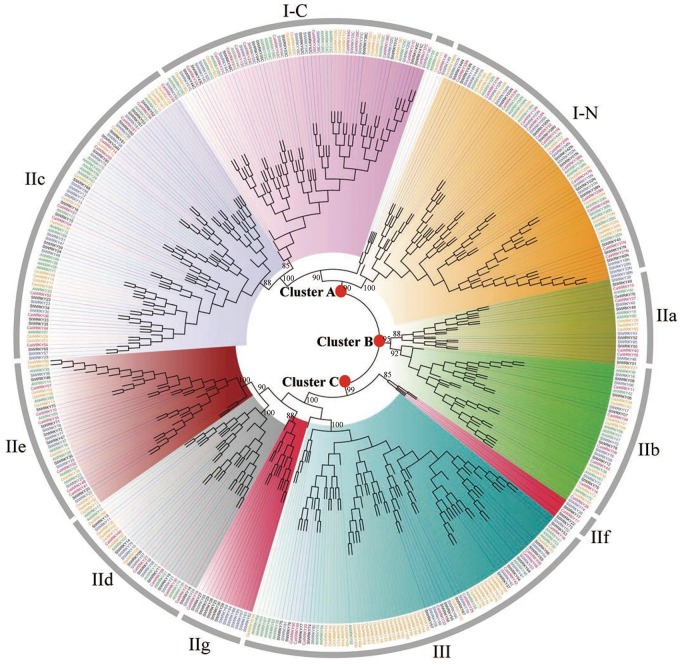
Phylogenetic tree of WRKY proteins from pepper, tomato, potato, Arabidopsis and rice using the neighbor joining method by MEGA 5.0. The WRKYs of each group were clustered together and labeled with different colors. The root nodes of Cluster A, Cluster B, and Cluster C were marked with red solid circles. Group IIf and IIg were two newly identified subgroups that only comprised WRKYs from Solanaceous plants. WRKYs from pepper (CaWRKY), tomato (SlWRKY), potato (StWRKY), Arabidopsis (AtWRKY), and rice (OsWRKY) are labeled in red, blue, black, green and yellow, respectively.

To better study the phylogenetic relationship of the 223 WRKYs in solanaceous plants, we constructed an unrooted phylogenetic tree based on the alignment of protein sequences of 444 complete WRKY domains (including N-terminal (I-N) and C-terminal (I-C) domains of group I) from pepper, tomato, potato, rice and Arabidopsis (Figure 1). Incomplete WRKY domains, including WRKY domains of CaWRKY01/CaWRKY12 in pepper, SlWRKY27/SlWRKY70 in tomato, and StWRKY21/StWRKY31/StWRKY32 in potato (Supplementary Table S1), were excluded from the phylogenetic analysis. Three main clusters including different WRKY groups were observed, which are cluster A (I-C+I-N+IIc), cluster B (IIa+IIb), and cluster C (IId+IIe+III+two outstanding expansions in group II) (Figure 1), respectively. In cluster A, the C-terminal WRKY domains of group I (I-C) and WRKY domains of group IIc seem to be more closely related on evolutionary level, and a unique expansion including five WRKY members (AtWRKY49, AtWRKY59, OsWRKY17, CaWRKY46, and SlWRKY49) was observed (Figure 1). In cluster C, two distinct gene expansion events adjacent to group IId+IIe and group III were identified, respectively. These two expansions were only composed of solanaceae WRKY members, and were tentatively defined as group IIf (CaWRKY17, SlWRKY26, StWRKY17, StWRKY22, and StWRKY73) and group IIg (CaWRKY19, SlWRKY62, SlWRKY63, SlWRKY64, SlWRKY65, SlWRKY66, SlWRKY67, SlWRKY68, StWRKY25, StWRKY26, StWRKY27, StWRKY28, StWRKY29, and StWRKY30) in this study (Figure 1 and Tables 1, 2). Eleven WRKYs were defined as None Group (NG) due to their incomplete WRKY domains (CaWRKY01, CaWRKY12, SlWRKY27, SlWRKY70, StWRKY21, StWRKY31, and StWRKY32) or phylogenetic uniqueness (CaWRKY46, CaWRKY52, SlWRKY49, and StWRKY45) (Figure 1 and Supplementary Table S1) (Huang et al., 2012; Huang and Liu, 2013).

### Group-Wise Structural Analysis of Solanaceae WRKY Genes

**FIGURE 2:**
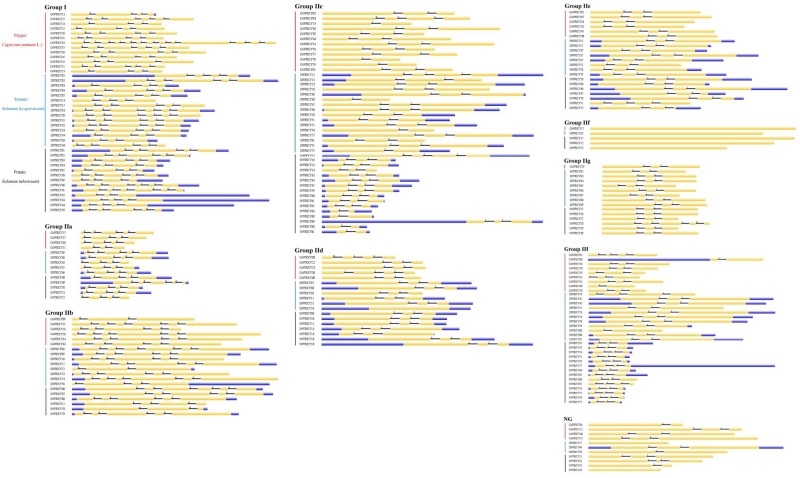
The group-wise analysis of *WRKY* gene structures from pepper (*Capsicum annuum*; CaWRKY), tomato (*Solanum lycopersicum*; SlWRKY), and potato (*Solanum tuberosum*; StWRKY). The upstream/downstream, exon and intron are shown with blue rectangle, yellow cylinder and black line, respectively. Intron phase numbers (0, 1, 2) are labeled on the top of each *WRKY* gene sketch map.

The structural analysis on 223 *WRKY* genes from pepper, tomato and potato were visualized using GSDS 2.0 in group-wise manner (Figure 2). Intron number of the *WRKY* genes demonstrated extensive variations, such as 0 (e.g., *StWRKY45*), 1 (e.g., *CaWRKY14*), 2 (e.g., *SlWRKY36*), 3 (e.g., *CaWRKY25*), 4 (e.g., *StWRKY38*), 5 (e.g., *CaWRKY51*), and 9 (*CaWRKY33*) (Supplementary Table S1). Nevertheless, a certain degree of conservatism was demonstrated by the dominated intron number of 2, which accounts for more than 50% (114/223) of the intron numbers in solanaceae *WRKY* genes. The intron number of different WRKY group members also displayed certain variations, as the group I (ranged from 2 to 5), group IIa (ranged from 2 to 4), and group IIb (ranged from 2 to 5) seemed to own relatively more introns than the other group members (ranged from 0 to 3) (Supplementary Table S1). The intron numbers are most variable among group I WRKY members (*CV* = 0.31), and relatively conserved among group IIa (*CV* = 0.17) and group IIe (*CV* = 0.11) WRKY members (Supplementary Table S1). Notably, *WRKY*s of group IIf (*CaWRKY17*, *SlWRKY26*, *StWRKY17*, *StWRKY22*, and *StWRKY73*) had distinct gene structure compared to the counterparts of other groups, due to their zero intron structure (Figure 2). A group-wise intron phase distribution was reflected by the intron phase profile analysis of the solanaceae *WRKY* genes, as all three different intron phases (0, 1, 2) could be detected in the *WRKY* genes of group I, IIc, IIg, III and NG, two intron phases (1 and 2) were found in group IId and IIe *WRKY*s, while only one intron phase (0) was detected in the *WRKY*s of group IIa and IIb. As for the intron phase pattern (pattern of intron phase constitution), three dominated (frequency > 70%) patterns were identified in some WRKY groups, including 0-0-0 pattern in group IIa (frequency of 71.4%), 2-2 pattern in group IId (frequency of 77.8%) and group IIe (frequency of 76.2%) (Supplementary Table S1). Thus, both the intron phase profile and intron phase pattern analysis suggest high genetic variations occurred in group I, IIc, IIg, III, and on the contrary, relatively genetic conservatism in group IIa, IId, and IIe (Figure 2 and Supplementary Table S1).

### Domain Composition and Conserved Amino Acids/Motif at the WRKY Domain Region of WRKY Proteins in Solanaceous Plants

**FIGURE 3:**
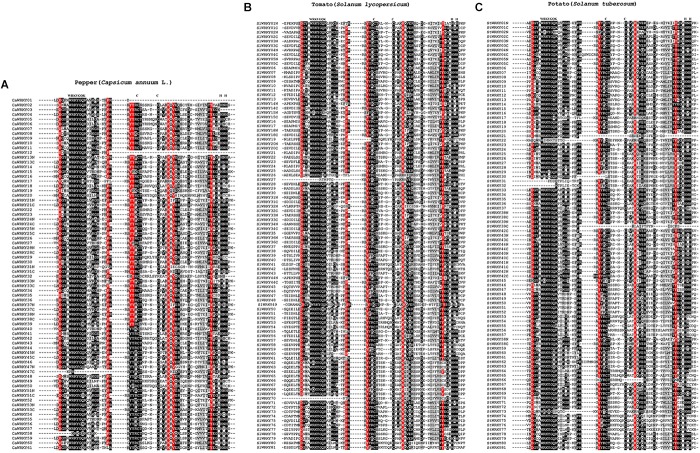
The sequence alignment of WRKY domains from solanaceous plants. **(A)** Pepper, **(B)** Tomato, and **(C)** Potato. The conserved amino acids were emphasized with black background. WRKYs from pepper, tomato and pepper were clustered together, respectively. The position of conserved heptapeptide (WRKYGQK) and zinc-finger consisting amino acids (C-C-H-H/C) were labeled on top. Amino acids with over 90% frequency were emphasized with red background.

As the most conserved structures of WRKY domain, the heptapeptide WRKYGQK and C_2_H_2_/C_2_HC zinc-finger structure are critical for DNA-binding and subsequent transcriptional regulation (Rushton et al., 1996, 2010). According to Figure 3, the WRKY domains of different solanaceous plants were relatively conservative as we expected. Nevertheless, some variations of the conserved heptapeptide WRKYGQK 7 structure, such as KKKGEK (CaWRKY58), WRKYGKK (SlWRKY60), and WHKYGQR (StWRKY55), were still detected. Except for the WRKYGQK polypeptide and Cys (C)/His (H) of zinc-finger, more highly conserved (occurrence rate ≥ 90%) amino acids (aa) were identified, including seven aa [Asp (D), Pro (P), Tyr (Y), Tyr (Y), Lys (K), Val (V) and Tyr (Y)] in pepper, five aa (D, P, Y, K and Y) in tomato, and four aa (D, Y, K and Y) in potato, respectively (Figure 3). Thus, the four common amino acids, D (4 amino acids pre-WRKYGQK), Y (3 amino acids pre C_1_), K (4 amino acids after C_2_) and Y (4 amino acids pre H_1_), are the most conserved amino acids of solanaceae WRKY domains, which may play conservative and important roles in the WRKY proteins of solanaceous plants.

**FIGURE 4:**
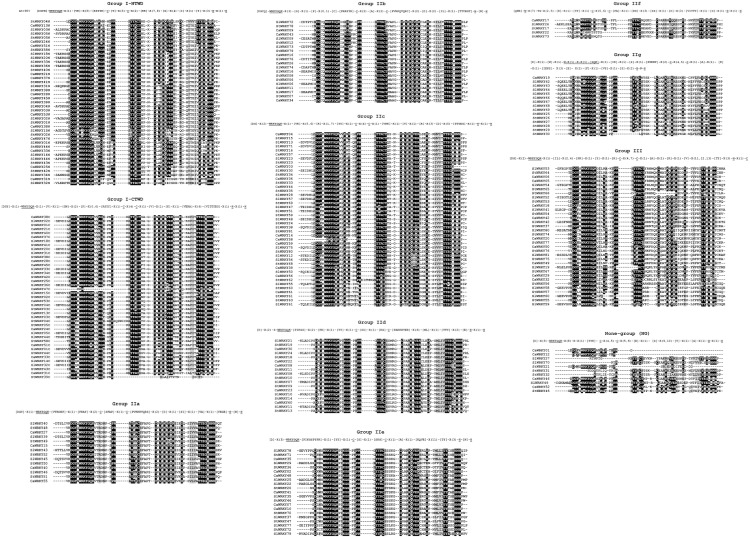
The group-wise sequence alignment of WRKY domains from solanaceous plants (pepper, tomato and potato). The group frames based on the conservatism of corresponding amino acids of WRKY groups were presented on top of each sequence alignment. The amino acids with occurrence rate over 90% were labeled in []; “X” indicate the random amino acids; The symbolic heptapeptide WRKYGQK and C_2_H_2_ of zinc-finger structure were annoted with underline.

The domain composition was further analyzed in a group-wise manner (Figure 4). The corresponding WRKY domain frame that recognizes WRKY domain sequences of each group was generated. For example, motif [DGY]-X(1)-WRKYGQK-[VTRDNP]-X(1)-[PRAY]-X(2)-C-[SFAP]-X(1)-C-[PVKKKVQRS]-X(2)-[D]-X(1)-[SI]-X(1)-[VA]-X(1)-[YEGE]-H-[N]-H was conducted to recognize all group IIa members (Supplementary Table S2). According to Figure 4, the heptapeptide WRKYGQK is extremely conserved among WRKY domains of group IIa, IIb, IId and IIe. On the contrary, the variations (WRKYGEN, WRKYGHK, WRKYGKK, WRKYGMK, etc.) were widely distributed in the WRKY domains of group IIc, IIf, IIg, and III. The WRKY domain frames exhibited different degrees of variations among different groups, as WRKY domains of group IIa, IIb, and IId own over 40 highly conserved amino acids (including WRKYGQK and C/H of zinc-finger), while the N-terminal of group I, group IIf and group III only have conserved amino acids of less than 30 (Supplementary Table S2). As for the detailed sequences of zinc-finger structures, the results showed that the variation of C_2_H_2_/C_2_HC zinc-finger structure was mainly occurred on the amino acid number between the first Cys (C_1_) and the second Cys (C_2_) or C_2_ and the first/only His (H_1_/H). The number of amino acids (4) between C_1_ and C_2_ of group I, as well as between C_2_ and H_1_ of group II (23) WRKY domains, were both consistent. On the contrary, the number of amino acids between C_1_ and C_2,_ as well as between C_2_ and H_1_ of group III WRKY domains seemed to be genetically inconstant (4 or 7 between C_1_ and C_2_; 22, 23, or 24 between C_2_ and H_1_) (Supplementary Table S2).

The group-wise sequence logo motif analysis further revealed the genetic variation situation of motifs in WRKY domain regions of each group (Supplementary Figure S1). As the motif of over five successive amino acids was defined as conserved motif in this study, group IIb has the most conserved motifs (WRKYGQK, CPRAYYR, PVRKQVQRC, and TTYEGT), while group III has no conserved motif at all (Supplementary Figure S1), indicating the occurrence of more intense variation in group III WRKY domains.

### Domain Composition Analysis in WRKY Proteins of Solanaceous Plants

Some functional domains except for the WRKY domain in solanaceae WRKYs were also detected, which were mainly existed in the WRKY members of group II, especially in the group IId members of pepper and group IIa/IIb/IId members of tomato and potato (Supplementary Table S3). Plant-ZN-ClUST (pfam10533) was the dominated domain of group IId WRKYs in pepper, tomato and potato, the function of the Plant-ZN-ClUST domain is still unclear. Some other common domains were also detected in the group IIa and group IIb WRKYs of tomato and potato. For instance, SlWRKY40 and StWRKY48 (group IIa members) both contain PHA03255 (BDLF3: PHA03255) and SSP160 (Special lobe-specific silk protein: pfam06933) domain. SlWRKY06, SlWRKY17, StWRKY06, and SlWRKY07 (group IIb members) all carried the common bZIP-Maf-small domain (Basic leucine zipper (bZIP) domain of small musculoaponeurotic fibrosarcoma (Maf) proteins: cd14717), which might be involved in various cell functions including proliferation, apoptosis, survival, and morphogenesis (Hang and Stein, 2011; Vanderford, 2011; Kannan et al., 2012). The larger average size of group IIb and IId WRKY proteins in tomato and potato might be caused by the extensively existence of extra domains (Table 1). No other domain except for WRKY was detected in group IIe, IIf, IIg, or III WRKY members of solanaceous plants (Supplementary Table S3). In summary, the domain composition of WRKYs that belong to different groups was also a reflection of genetic conservatism in group IIa, IIb, and IId, and genetic variation in group IIe, IIf, IIg, and III, as the WRKY groups with more common domains tend to be more genetic and functionally conserved.

### Orthologue Analysis of WRKY Genes in Solanaceous Plants

**FIGURE 5:**
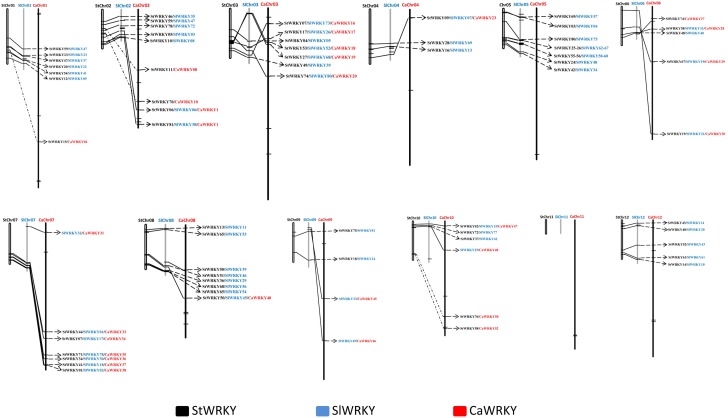
Orthologue analysis of *WRKY* genes in potato (*Solanum tuberosum*), tomato (*Solanum lycopersicum*) and pepper (*C. annuum*). The white, gray and black columns represent the chromosomes that belong to potato, tomato, and pepper, respectively. The StWRKYs, SlWRKYs, and CaWRKYs were labeled in black, blue and red, respectively. The orthologures of StWRKY, SlWRKY, and CaWRKY were connected with black lines.

A Best-BLAST approach was employed to identify candidate StWRKY/SlWRKY/CaWRKY orthologies among potato, tomato and pepper. In defining probable orthologous pairs (two WRKYs) or combinations (three WRKYs), we required that orthologous proteins belong to the same structural class (based on phylogenetic tree) (Figure 1) and have a pairwise identity of over 90% (based on sequence blast). Corresponding candidate orthologous pairs or combinations were also filtered by chromosome localization (Grube et al., 2000; Xu et al., 2016). Totally 119 WRKY protein pairs were detected as bona fide orthologs, labeled on the corresponding chromosomes, and linked with lines to highlight the relative positions in their genomes (Figure 5). Of the 119 WRKY orthologous pairs identified, nearly 60% (71) of which were SlWRKY/StWRKY pairs, compared to only 23 SlWRKY/CaWRKY and 25 StWRKY/CaWRKY pairs, respectively (Figure 5), which confirmed the notable variation between the *Capsicum* genus (pepper) and the *Solanum* genus (tomato and potato). The 19 orthologous combinations were identified from seven different chromosomes, as chromosome 02 (2), chromosome 03 (5), chromosome 04 (1), chromosome 06 (3), chromosome 07 (6), chromosome 08 (1), and chromosome 10 (1), indicating the relatively lower degree of genetic variation of *WRKY*s in these chromosomes, especially in chromosome 03 and chromosome 07 (Figure 5).

### Expression Analysis of CaWRKY Genes in Various Pepper Tissues or Under Different Biotic/Abiotic Stresses

**FIGURE 6:**
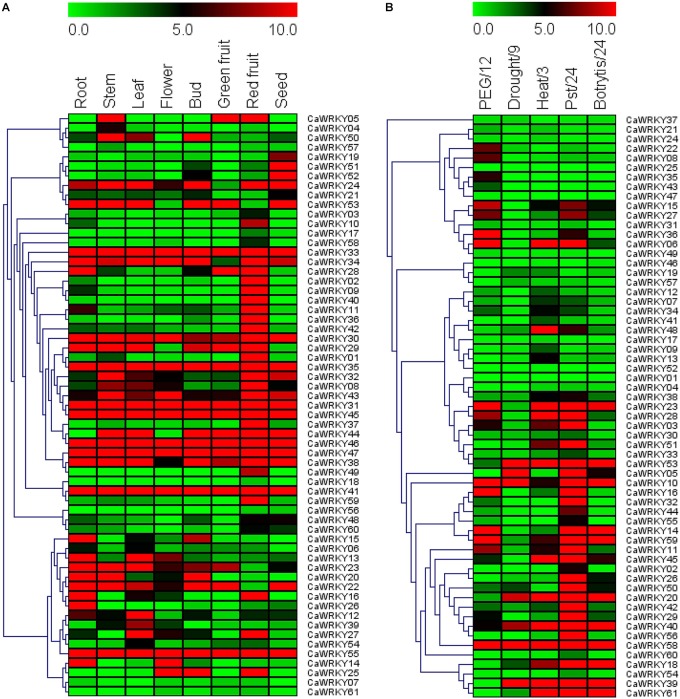
Expression profiles of 61 *CaWRKY* genes in various pepper tissues **(A)** and under different biotic/abiotic stresses **(B)**. **(A)** *CaWRKY* gene expression was determined by RT-qPCR. *CaWRKY*s are indicated as rows and tissues as columns. Green, black and red elements represented down-regulated, no change and up-regulated, respectively. **(B)** *CaWRKY* gene expression was determined by RT-qPCR. *CaWRKY*s are represented as rows and stress treatment/time point as columns (PEG/12: 20% PEG6000 for 12 h; Drought/9: Water withheld for 9 days; Heat: 42°C for 3 h; Pst/24: *Pst*DC3000 infection for 24 h; Botrytis/24: *Botrytis cinerea* infection for 24 h. The vertical dendrograms represent the similarity degree of tissue expression profile **(A)** and stress response profile **(B)** among the 61 *CaWRKY* genes.

To explore the functional diversity of solanaceae WRKY proteins, we analyzed the tissue expression pattern of the 61 *CaWRKY* genes by RT-qPCR (Figure 6A). Three representative tissue expression patterns of the *CaWRKY*s were demonstrated, including constitutive expression (expressed in all tested tissues), low expression (barely expressed in any of the tissue tested), specific/preferential expression (significantly high expression in a specific tissue). According to Figure 6A, half of the constitutively expressed *CaWRKY* genes [*CaWRKY30* (IId), *CaWRKY31* (I), *CaWRKY33* (I), *CaWRKY35* (IIe), *CaWRKY38* (I), *CaWRKY41* (IIe), *CaWRKY45* (I), *CaWRKY46* (NG), *CaWRKY47* (I), and *CaWRKY55* (IIa)] belong to group I, and none belongs to group III *CaWRKY* in this expression pattern. On the contrary, thirteen *CaWRKY* genes including *CaWRKY03* (IIc), *CaWRKY04* (IIc), *CaWRKY06* (III), *CaWRKY07* (IIe), *CaWRKY17* (IIf), *CaWRKY18* (III), *CaWRKY48* (IIe), *CaWRKY54* (IIc), *CaWRKY56* (IIc), *CaWRKY57* (IIc), *CaWRKY58* (IIc), *CaWRKY60* (IId), and *CaWRKY61* (IIc), were found to be barely expressed in any of the tissue tested (Figure 6A). Group IIc *CaWRKY*s was the dominated (7/13) members of the low expression pattern, and none of group I *CaWRKY* was detected in this pattern. As for the specific/preferential expression pattern, relatively more *CaWRKY* genes including seed specifically/preferentially expressed *CaWRK*s [*CaWRKY19* (IIf), *CaWRKY51* (I), *CaWRKY52* (NG)], leaf specifically/preferentially expressed *CaWRKY*s [*CaWRKY12* (NG), *CaWRKY39* (IIc)], root specifically/preferentially expressed *CaWRKY*s [*CaWRKY15* (IIa), *CaWRKY26* (IIb)], stem specifically/preferentially expressed *CaWRKY*04 (IIc), and full ripening fruit specifically/preferentially expressed *CaWRKY*s [*CaWRKY01* (NG), *CaWRKY02* (IIe), *CaWRKY09* (IIb), *CaWRKY10* (IIe), *CaWRKY11* (IIb), *CaWRKY36* (IIc), *CaWRKY37* (I), *CaWRKY40* (IIa), *CaWRKY42* (III), *CaWRKY49* (III), and *CaWRKY59* (IIc)], were respectively identified (Figure 6A). The remaining *CaWRKYs* were expressed in two or more different tissues with diversified expression patterns (Figure 6A).

In addition to biotic stress, abiotic stresses, such as drought, salinity and extreme temperatures are important factors that constrain agricultural productivity worldwide (Chen et al., 2013; Wang et al., 2016; Li et al., 2018). According to the stress response pattern of *CaWRKY*s reflected in Figure 6B, more than half (32/61) of *CaWRKY* genes were significantly induced by one or more biotic/abiotic stresses tested (osmotic stress, drought, heat, *Pst*DC3000 and *B. cinerea*). Overall, compared to abiotic stresses (PEG, drought and heat), *CaWRKY*s were more sensitive to pathogen (*Pst*DC3000 and *B. cinerea*) infections. For example, 25 *CaWRKY* genes were significantly up-regulated under *Pst*DC3000 infection, and eleven *CaWRKY*s were induced by *B. cinerea*. Meanwhile, only eight PEG-induced, five drought-induced and eleven heat-induced *CaWRKY* genes were detected. It is worth mentioning that nearly 70% (7/13) of the group I *CaWRKY*s seemed to be insensitive to any of the stress imposed, while nearly 90% (8/9) of the group III *CaWRKY*s (*CaWRKY49*) was induced by at least one of the stresses tested.

### Functional Analyses Suggested Differential Roles of CaWRKYs in Disease Resistance and Abiotic Stress Responses in Pepper

**FIGURE 7:**
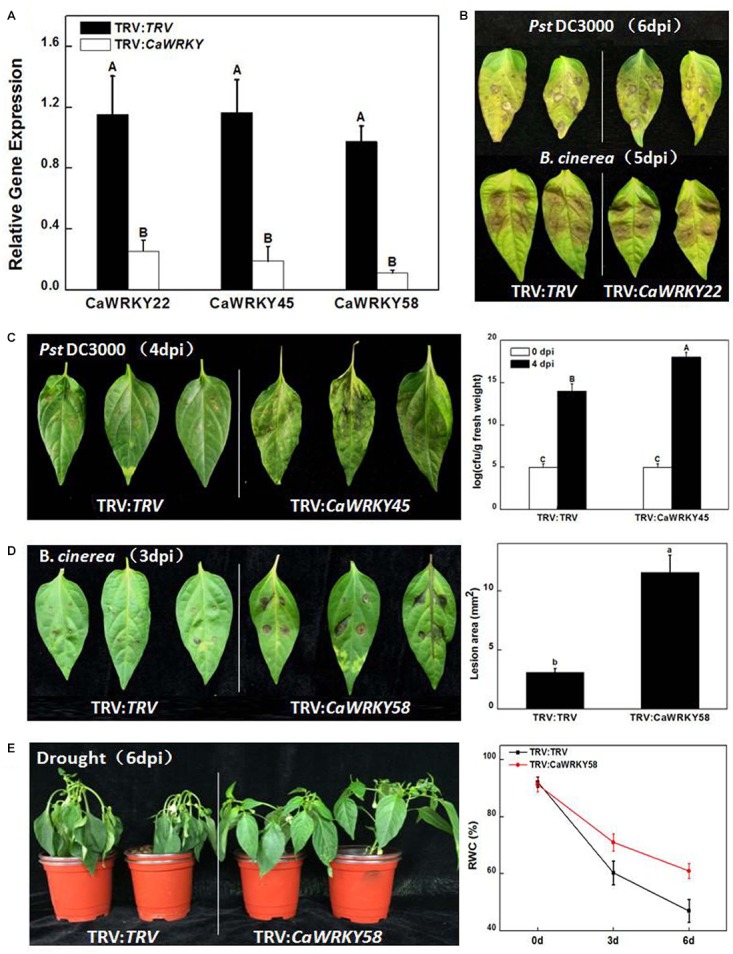
Functions of the *CaWRKY* genes in disease resistance and abiotic stress responses indicated by Virus induced gene silencing (VIGS). **(A)** Silencing efficiency. Pepper plants infiltrated with *Agrobacterium* suspensions carrying an empty pTRV vector served as control plants. Expression levels of *CaWRKY*s in control and VIGS-treated plants were detected by RT-qPCR assays. **(B)** *Pst*DC3000 (Up) and *B. cinerea* (down) infections. Photographs were taken at 6 dpi days past inoculation) and 5 dpi, respectively. **(C)** Enhanced susceptibility to *Pst*DC3000. pTRV-control plants (TRV:TRV) and *CaWRKY45*-silenced plants (TRV:*CaWRKY45*) were inoculated with *Pst*DC3000 and the picture was taken at 4 dpi (Left). According to DMRT (Duncan’s multiple range test), means of the colony-forming units (cfu) in leaves of TRV:*CaWRKY45* is significantly higher than control at 4 dpi (*P* < 0.01) (Right). **(D)** Enhanced susceptibility to *B. cinerea*. TRV:TRV and TRV:*CaWRKY58* plants were inoculated with *B. cinerea* and the photograph was taken at 3 dpi (Left). Lesion diameter was measured and statistically calculated for all plants. Significant difference between lesion diameters of the silenced plants and that of the control plants is indicated at *P* < 0.05 according to DMRT (Right). **(E)** Drought stress response. Phenotypes of the *CaWRKY58*-silenced and control plants after withholding water for 6 days (Left). Comparisons of TRV:CaWRKY58 and TRV:TRV control plants at 6th day after drought treatment. Twelve plants were used for each treatment. The experiments were conducted three times independently. Values represent mean ± SD (*n* = 3).

To directly analyze the biological functions of *CaWRKY* genes from pepper, we first screened for the 32 *CaWRKY* genes that were induced by at least one of the stresses tested (Figure 7B), and obtained the corresponding gene silencing lines through Virus induced gene silence (VIGS) technology. The resistance of the 32 *CaWRKY* gene silenced plants to *Pst*DC3000, *B. cinerea* and drought were tested, and several representative phenotypes determined by the silencing of some *CaWRKY* genes were detected in this study (Figure 7).

The silencing effects were partially demonstrated in Figure 7A, from which the reduced expression patterns of *CaWRKY22*, *CaWRKY45*, and *CaWRKY58* were evident. Figure 7B was intended as negative control, showing that most silencing plants had no phenotype, just like *CaWRKY22* silenced plants. *CaWRKY45* silenced plants showed compromised resistance to *Pst*DC3000 (Figure 7C). Disease lesions developed quicker and stronger in *CaWRKY45* silenced plants and the bacterial growth was about 5-log higher. Consistently, the expression of *CaWRKY45* was highly induced by *Pst*DC3000, but not *B. cinerea*. *CaWRKY45* was consistently expressed in all pepper tissues detected (Figure 6A), which may point to a life-keeping as well as defense-related role of this important WRKY protein. Another gene, *CaWRKY58*, showed reduced resistance to *B. cinerea*. As shown in Figure 7D, both images and measurement demonstrated significantly increased lesion area caused by *B. cinerea* at 3 days post-inoculation in *CaWRKY58*-silenced plants. In contrast to *CaWRKY45*, *CaWRKY58* was expressed at very low level in most tissues (except in red fruit), and was induced by all stresses (both biotic and abiotic). Thus, CaWRKY58 may be purely stress-responsive or stress-related protein. Interestingly, the *CaWRKY58*-silenced plants were also more tolerant to drought stress (Figure 7E).

## Discussion

### Extensive Genetic Variations Occur in Solanaceae WRKYs

With the advances in genome sequencing technology, the whole genome sequences of tomato, potato, and pepper were subsequently accomplished (The Tomato Genome Consortium, 2012; Qin et al., 2014), and the corresponding WRKY TF families of these three plants were unveiled in recent years (Huang et al., 2012; Huang and Liu, 2013; Cheng et al., 2016). Here, a comparative analysis was conducted on the WRKY families of pepper, tomato and potato, and extensive genetic variations of the solanaceae WRKYs were demonstrated by the variations of WRKY number/size, group constitution, phylogenetic relationship, gene structure, and domain composition. Moreover, significant genetic variations were observed between the *Capsicum* genus (pepper) and the *Solanum* genus (tomato, potato) (Figure 5 and Tables 1, 2), and some minor variations on WRKY group constitution and average WRKY size of each group were also detected between tomato and potato that both belong to the *Solanum* genus (Tables 1, 2).

Many genetic variations were revealed in group-wise manner. For example, *WRKY* genes of group I were most variable on the gene structure characteristics (intron number, intron phase profile, intron phase pattern) among all solanaceae WRKY groups (Figure 2 and Supplementary Table S1). The group II (especially group IIc and IIg) was the main group in which the *WRKY* gene loss/gain variations occurred in solanaceous plants (Table 1). The group III WRKYs experienced the most intense genetic variation during evolutionary process, revealed by the least number of conserved amino acids (only 24), zero number of motif in WRKY domain, and highly variant zinc-finger structure of this group members (Supplementary Figure S1 and Supplementary Table S2). Moreover, the only existence of WRKY domain (no other common functional domain detected) in group III WRKY members was also an indication of high variability of this group (Supplementary Table S3). As for the solanaceae specific WRKY groups including group IIf and IIg (Figure 1), group IIf had the unique gene structure of no intron and relatively smaller size (average size of 690 bp in solanaceae, average size of 786 bp in pepper, average size of 660 bp in tomato, average size of 668 bp in potato) (Figure 2 and Table 2), and group IIg was the main group in which variation in WRKY number occurred between the *Capsicum* genus and *Solanum* genus (Table 1). Considering these distinctive characteristics, further analysis is required to elucidate the evolutionary specificity of these two solanaceae-specific WRKY groups in the future.

It is well accepted that the existence of almost invariable WRKYGQK motif and C_2_H_2_/C_2_HC zinc-finger is the symbolic structure of WRKY domains (Eulgem et al., 2000; Xie et al., 2005), which are necessary but not sufficient to judge a WRKY. Previous studies have shown that WRKYGQK is not completely invariable (Xie et al., 2005; Huang et al., 2012; Xu et al., 2016), and some TFs other than WRKY also contain zinc-finger structures (Babu et al., 2006; Duan et al., 2007; Shi et al., 2014). Here, various WRKYGQK variants in WRKYs of solanaceous plants were identified, more than half of them were from potato (Figure 3), and the remaining variants were mainly distributed across the solanaceae WRKY domains of group IIc, IIf, IIg and III (Figure 4). As for zinc-finger, our group-wise frame analysis (Supplementary Table S2) showed a variant zinc-finger structure (CX_4,7_CX_23_HXC) of group III in solanaceae WRKYs, which differs from the well recognized CX_7_CX_23_HXC zinc-finger structure (Rushton et al., 2010; Cheng et al., 2012).

### Certain Degrees of Genetic Conservatism Present in Solanaceae WRKYs

Although this study was mainly focused on the genetic variation analysis, certain extent of conservatism in the solanaceae WRKYs was also demonstrated in this study. The most direct evidence was the existence of four common conserved amino acids in the solanaceae WRKY domains, which are the D (4 amino acids pre-WRKYGQK), Y (3 amino acids pre the first C of zinc finger), K (4 amino acids after the second C of zinc finger), and Y (4 amino acids pre the first/only H of zinc finger) (Figure 3). Moreover, the analysis of WRKY number, group constitution and average WRKY size, domain composition and ortholog pairs showed certain degree of genetic conservatism between the WRKYs of tomato and potato (Figure 1–5 and Tables 1, 2). The group-wise analysis of *WRKY* gene structure features (intron number, intron phase, intron phase pattern) showed that group IIa and IIe were relatively more conserved compared to *WRKY* genes of other groups, determined by their lower CV of intron number, simpler composition of intron phase (0, 2) and corresponding intron phase pattern (0-0-0, 2-2) (Supplementary Table S1).

### Gene Expression Profile Analysis of Pepper *CaWRKY*s

Although the full genome sequence of pepper was recently completed (Qin et al., 2014), no systematic research on CaWRKYs of pepper had been conducted so far. Herein, we concluded that the constitutively expressed *CaWRKY*s (10 members) and low expressed *CaWRKY*s (13 members) both possessed relatively low percentage of the whole *CaWRKY* gene family, which revealed the potential functional diversity of *CaWRKY*s in pepper. The group-wise analysis showed that the *CaWRKY*s of group I were the main part of constitutive expression pattern members (50%), while no group I *CaWRKY* was detected in the low expression pattern members. Furthermore, *CaWRKY*s of group IIc seemed to be the dominant members in low expression pattern, and no group III *CaWRKY* was found to be constitutively expressed in all the tested tissues. As for the specifical/preferential expressed pattern, *CaWRKY*s that belong to various groups were included, and no special distribution role was detected. Nevertheless, these specific/preferentially expressed *CaWRKY*s could be related to the particular character or functions of the tissues where they expressed. To date, most studies on WRKY TFs were focused on their resistance functions, and plant leaves were always collected for relative research. Our study showed the possibility that *CaWRKYs* may play corresponding roles in the development of various pepper organs, especially fruit maturation, as a large number of *CaWRKY*s were specifically expressed in the red fruits in general. According to the responsiveness of *CaWRKY*s to various biotic and abiotic stressors, more than 50% of *CaWRKY* genes were sensitive to one or more biotic/abiotic stresses tested (osmotic stress, drought, heat, *Pst*DC3000 and *B. cinerea*). Nevertheless, nearly 70% of group I *CaWRKY*s seemed to be insensitive to any of the stress used, on the contrary, nearly 90% of group III *CaWRKY*s was induced by at least one of the stresses imposed, which indicated a high activity of stress response of group III. As both the tissue expression and stress response pattern of *CaWRKY*s were somehow correlated with their group classification, we propose that WRKY TFs may play their specific biological roles in group-wise manner, which is worthy a lot of subsequent study.

### Some Pepper CaWRKYs Play Critical Roles in Disease Resistance and Abiotic Stress Responses

Before the release of PGD (Release 2.0^1^), eight WRKY protein encoding genes from pepper had been reported to play critical roles in biological processes, including pathogen resistance and high-temperature tolerance (Dang et al., 2013, 2014; Cai et al., 2015; Xu et al., 2016). Consistent with earlier studies, *CaWRKY10* (previously named *CaWRKY27*) was induced by most stresses except heat, while CaWRKY15 was induced by PEG and *Pst*DC3000 and *CaWRKY34* (previously named *CaWRKY6*) was induced by heat stress. *CaWRKY45* (named *CaWRKY-a*) was induced by both heat and *Pst*DC3000. Interestingly, *CaWRKY58* (previously named as *CaWRKY-b*) was “most stress responsive,” as its tissue-specific expressions were low in general, but its stress-responsive expressions were high in all stresses tested. Nevertheless, the function study of CaWRKY proteins in pepper was still very limited, compared to Arabidopsis, rice and tomato. Just like its counterparts in other plants, CaWRKYs seem to be closely involved in various stress responsive processes, revealed by the drastic expression changes of most *CaWRKY* genes under two disease infections (*Pst*DC3000 and *B. cinerea*) or three abiotic stresses (osmotic stress, drought and high temperature). From a functional point of view, our VIGS experiments demonstrated a positive role of CaWRKY58 in disease resistance (*B. cinerea*) and a negative role in drought tolerance. This WRKY protein can be multi-functional in stress responses, and its role in other stresses and the correlation and connection of all those stress responsive pathways are the appealing research directions for the future. What’s more, CaWRKY58 might have multiple interacting partners to carry out all its functions in various stresses. CaWKRY45 was shown by VIGS to positively regulate *Pst*DC3000 defense pathways. This phenotype fitted with the *Pst*DC3000 responsive expression of *Ca*WRKY45. In support to our findings, earlier publication (using the name CaWRKY-a) suggested that *TMV*, *Xcv* and salicylic acid (SA)-induced expression of *CaWRKY45*. It will be interesting to further identify its interacting partners and find out the affected pathways (either PTI or ETI) of CaWRKY45 in pepper. It is surprising that many defense responsive *CaWRKY* genes showed no phenotype in response to the stresses we tested. Perhaps, the limitations of VIGS or the gene redundancy may hide the phenotype in silenced plants in our study. However, the currently discovered phenotypes shed some light on the functionality of the CaWRKY family, and further researches will be continued to unravel the underlining mechanisms.

## Conclusion

The WRKY gene family has been demonstrated to be involved in various biological processes including plant development and responses to biotic/abiotic stresses (Pandey and Somssich, 2009; Li et al., 2013a; Dang et al., 2014; Cai et al., 2015). The comprehensive and systematic comparative analysis of 223 WRKY members from solanaceae crops (pepper, tomato and potato) demonstrated tremendous genetic variations among WRKY members of different solanaceous plants or groups, as well as certain degrees of conservatism for some solanaceae WRKYs. Moreover, the expression analysis and functional exploration of CaWRKYs in pepper provide insight into the functional divergence of the WRKY gene family from pepper. The results of bioinformatics and functional analysis might provide basic resources for further dissection of the evolutionary clues of WRKYs in solanaceae crop plants, and also elucidation of the functional diversity of CaWRKYs in pepper.

## Author Contributions

HW and GZ supervised and designed the experiments. YC, QY, ZY, and MR performed the experiments. YC and GA conceived the project and wrote the manuscript with contributions from ZL and RW.

## Conflict of Interest Statement

The authors declare that the research was conducted in the absence of any commercial or financial relationships that could be construed as a potential conflict of interest.

## Footnotes

1 http://pepperhub.hzau.edu.cn

2 https://solgenomics.net/

3 http://potatogenome.berkeley.edu/nsf5

4 http://pfam.xfam.org/

5 http://www.clustal.org/

6 http://gsds.cbi.pku.edu.cn

7 http://smart.emblheidelberg.de/

8 http://weblogo.berkeley.edu/

9 http://www.ncbi.nlm.nih.gov/cdd

10 http://chibba.agtec.uga.edu/duplication/index/locus

11 www.genscript.com

12 http://vigs.solgenomics.net/

## Supplementary Material

FIGURE S1 The group-wise logo motif analysis of WRKY domains from solanaceous plants (pepper, tomato and potato). The bit score for each position in the sequence and the group names are indicated to the left. The blue solid circle and brown solid square label the conserved heptapeptide and zinc-ginger structure, respectively. The green triangle below the logo indicates the distinct motif uniquely existing in the corresponding group. The red asterisks below indicate the highly conserved amino acids in each group.

TABLE S1 The WRKY intron features of solanaceous plants.

TABLE S2 The group-wise analysis of WRKY domain frame, conserved amino acids number and zinc-finger structure.

TABLE S3 Domain composition analysis in WRKY proteins of solanaceous plants.

TABLE S4 Primers of *CaWRKY* genes for RT-qPCR analysis.

TABLE S5 Primers of *CaWRKY* genes used for VIGS analysis.
