# Supplementary figures and images for: Comparative Genomic Analysis Reveals Extensive Genetic Variations of WRKYs in Solanaceae and Functional Variations of CaWRKYs in Pepper
Source: Front Genet. 2019 May 28;10:492. doi: 10.3389/fgene.2019.00492 (PMC6546733; doi:10.3389/fgene.2019.00492)

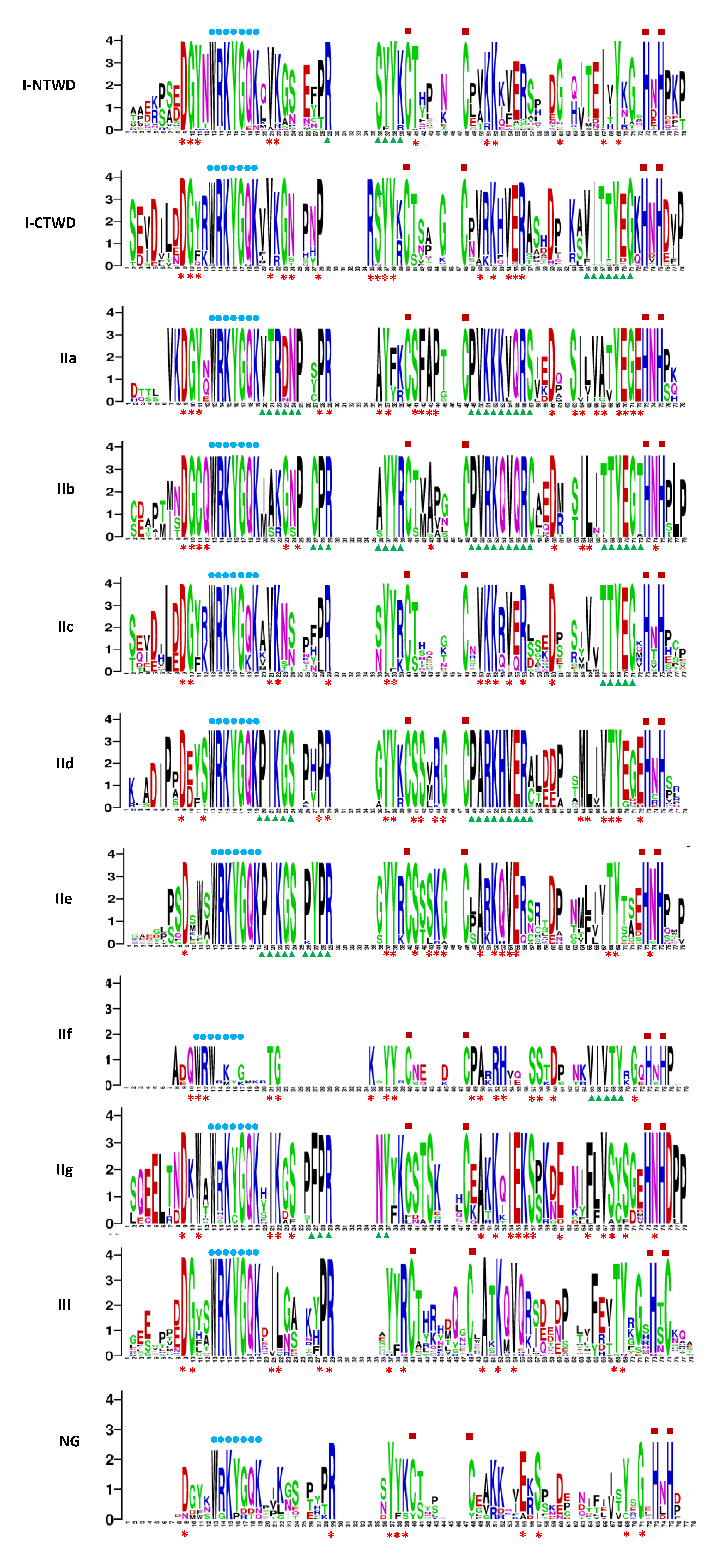

Supplement: FIGURE S1 — The group-wise logo motif analysis of WRKY domains from solanaceous plants (pepper, tomato and potato). The bit score for each position in the sequence and the group names are indicated to the left. The blue solid circle and brown solid square label the conserved heptapeptide and zinc-ginger structure, respectively. The green triangle below the logo indicates the distinct motif uniquely existing in the corresponding group. The red asterisks below indicate the highly conserved amino acids in each group. [file Image_1.TIFF]
