# Supplementary material for: Comparative Genomic Analysis Reveals Extensive Genetic Variations of WRKYs in Solanaceae and Functional Variations of CaWRKYs in Pepper
Source: Front Genet. 2019 May 28;10:492. doi: 10.3389/fgene.2019.00492 (PMC6546733; doi:10.3389/fgene.2019.00492)
Supplement: TABLE S1 — The WRKY intron features of solanaceous plants. [file Table_1.DOCX]

Supplementary Table S1 The WRKY intron features of solanaceous plants

| **Group** | **Gene symbol** | **Protein ID** | **Intron No** | **Intron phase pattern** | **Dominated phase patterns (%)** | | |
| --- | --- | --- | --- | --- | --- | --- | --- |
| I | CaWRKY13 | Capana02g003339 | 3 | 0 2 2 | 0 2 2  （11/43）  （25.6%） | 0 0 2 2  (10/43)（23.3%） | 0 0 1 2 2（6/43）（13.95%） |
|  | CaWRKY21 | Capana03g003085 | 5 | 0 0 1 2 2 |  |  |  |
|  | CaWRKY24 | Capana04g001820 | 3 | 0 2 2 |  |  |  |
|  | CaWRKY25 | Capana05g002502 | 3 | 0 2 2 |  |  |  |
|  | CaWRKY28 | Capana06g001506 | 4 | 0 0 2 2 |  |  |  |
|  | CaWRKY31 | Capana07g000181 | 4 | 1 0 2 2 |  |  |  |
|  | CaWRKY33 | Capana07g001256 | 9 | 020201221122 |  |  |  |
|  | CaWRKY37 | Capana07g002350 | 5 | 0 0 1 2 2 |  |  |  |
|  | CaWRKY38 | Capana07g002454 | 4 | 0 0 2 2 |  |  |  |
|  | CaWRKY45 | Capana09g001251 | 4 | 0 0 2 2 |  |  |  |
|  | CaWRKY47 | Capana10g000205 | 3 | 0 0 2 |  |  |  |
|  | CaWRKY51 | Capana10g001791 | 5 | 0 0 1 2 2 |  |  |  |
|  | CaWRKY53 | Capana11g001882 | 3 | 0 2 2 |  |  |  |
|  | SlWRKY01 | solyc07g047960.2.1 | 3 | 1 2 2 |  |  |  |
|  | SlWRKY02 | solyc07g066220.2.1 | 4 | 0 0 2 2 |  |  |  |
|  | SlWRKY03 | solyc02g088340.2.1 | 3 | 0 2 2 |  |  |  |
|  | SlWRKY04 | solyc05g012770.2.1 | 3 | 0 2 2 |  |  |  |
|  | SlWRKY05 | solyc03g104810.2.1 | 3 | 0 2 2 |  |  |  |
|  | SlWRKY14 | solyc12g006170.1.1 | 3 | 1 2 2 |  |  |  |
|  | SlWRKY15 | solyc10g005680.1.1 | 4 | 0 0 2 2 |  |  |  |
|  | SlWRKY18 | solyc07g065260.2.1 | 5 | 0 0 1 2 2 |  |  |  |
|  | SlWRKY20 | solyc12g014610.1.1 | 5 | 0 0 1 2 2 |  |  |  |
|  | SlWRKY31 | solyc06g066370.2.1 | 4 | 0 0 2 2 |  |  |  |
|  | SlWRKY32 | solyc07g005650.2.1 | 4 | 1 0 1 2 |  |  |  |
|  | SlWRKY33 | solyc09g014990.2.1 | 4 | 0 0 2 2 |  |  |  |
|  | SlWRKY34 | solyc05g055750.2.1 | 3 | 0 2 2 |  |  |  |
|  | SlWRKY36 | solyc04g056360.2.1 | 2 | 2 2 |  |  |  |
|  | SlWRKY44 | solyc10g084380.1.1 | 4 | 0 0 2 2 |  |  |  |
|  | StWRKY01 | PGSC0003DMP400038283 | 4 | 0 0 2 2 |  |  |  |
|  | StWRKY02 | PGSC0003DMP400019959 | 4 | 1 1 0 0 |  |  |  |
|  | StWRKY03 | PGSC0003DMP400049274 | 3 | 0 2 2 |  |  |  |
|  | StWRKY04 | PGSC0003DMP400009822 | 3 | 1 1 0 |  |  |  |
|  | StWRKY05 | PGSC0003DMP400002598 | 3 | 0 2 2 |  |  |  |
|  | StWRKY38 | PGSC0003DMP400029302 | 4 | 0 0 2 2 |  |  |  |
|  | StWRKY39 | PGSC0003DMP400020631 | 3 | 0 0 2 |  |  |  |
|  | StWRKY40 | PGSC0003DMP400051947 | 5 | 0 0 1 2 2 |  |  |  |
|  | StWRKY41 | PGSC0003DMP400038615 | 5 | 1 1 2 0 0 |  |  |  |
|  | StWRKY42 | PGSC0003DMP400040389 | 2 | 2 2 |  |  |  |
|  | StWRKY43 | PGSC0003DMP400050209 | 3 | 1 1 2 |  |  |  |
|  | StWRKY44 | PGSC0003DMP400010920 | 3 | 1 1 2 |  |  |  |
|  | StWRKY58 | PGSC0003DMP400019408 | 4 | 1 1 0 0 |  |  |  |
|  |  |  | CV=0.31 |  |  |  |  |
| II a | CaWRKY15 | Capana03g000473 | 4 | 0 0 0 0 | 0 0 0  (10/14)（71.4%） | 0 0 0 0（3/14）（21.4%） | 0 0  （1/14）（7.1%） |
|  | CaWRKY27 | Capana06g001110 | 3 | 0 0 0 |  |  |  |
|  | CaWRKY40 | Capana08g000683 | 3 | 0 0 0 |  |  |  |
|  | CaWRKY55 | Capana12g001134 | 2 | 0 0 |  |  |  |
|  | SlWRKY39 | solyc03g116890.2.1 | 4 | 0 0 0 0 |  |  |  |
|  | SlWRKY40 | solyc06g068460.2.1 | 3 | 0 0 0 |  |  |  |
|  | SlWRKY43 | solyc12g042590.1.1 | 3 | 0 0 0 |  |  |  |
|  | SlWRKY45 | solyc08g067360.2.1 | 3 | 0 0 0 |  |  |  |
|  | SlWRKY46 | solyc08g067340.2.1 | 3 | 0 0 0 |  |  |  |
|  | StWRKY48 | PGSC0003DMP400049656 | 3 | 0 0 0 |  |  |  |
|  | StWRKY49 | PGSC0003DMP400034429 | 4 | 0 0 0 0 |  |  |  |
|  | StWRKY50 | PGSC0003DMP400013078 | 3 | 0 0 0 |  |  |  |
|  | StWRKY51 | PGSC0003DMP400013081 | 3 | 0 0 0 |  |  |  |
|  | StWRKY52 | PGSC0003DMP400049886 | 3 | 0 0 0 |  |  |  |
|  |  |  | CV=0.17 |  |  |  |  |
| II b | CaWRKY09 | Capana02g000918 | 2 | 0 0 | 0 0 0 0（10/20）（50%） | 0 0 0 0 0（5/20）（25%） | 0 0  （3/20）（15%） |
|  | CaWRKY11 | Capana02g002230 | 5 | 0 0 0 0 0 |  |  |  |
|  | CaWRKY16 | Capana03g001099 | 4 | 0 0 0 0 |  |  |  |
|  | CaWRKY26 | Capana06g001008 | 5 | 0 0 0 0 0 |  |  |  |
|  | CaWRKY34 | Capana07g001387 | 4 | 0 0 0 0 |  |  |  |
|  | CaWRKY43 | Capana08g001961 | 4 | 0 0 0 0 |  |  |  |
|  | SlWRKY06 | SlWRKY06 | 5 | 0 0 0 0 0 |  |  |  |
|  | SlWRKY09 | SlWRKY09 | 4 | 0 0 0 0 |  |  |  |
|  | SlWRKY16 | SlWRKY16 | 4 | 0 0 0 0 |  |  |  |
|  | SlWRKY17 | SlWRKY17 | 4 | 0 0 0 0 |  |  |  |
|  | SlWRKY72 | SlWRKY72 | 2 | 0 0 |  |  |  |
|  | SlWRKY73 | SlWRKY73 | 4 | 0 0 0 0 |  |  |  |
|  | SlWRKY74 | SlWRKY74 | 5 | 0 0 0 0 0 |  |  |  |
|  | SlWRKY76 | SlWRKY76 | 3 | 0 0 0 |  |  |  |
|  | StWRKY06 | StWRKY06 | 5 | 0 0 0 0 0 |  |  |  |
|  | StWRKY07 | StWRKY07 | 4 | 0 0 0 0 |  |  |  |
|  | StWRKY08 | StWRKY08 | 4 | 0 0 0 0 |  |  |  |
|  | StWRKY12 | StWRKY12 | 3 | 0 0 0 |  |  |  |
|  | StWRKY78 | StWRKY78 | 2 | 0 0 |  |  |  |
|  | StWRKY79 | StWRKY79 | 4 | 0 0 0 0 |  |  |  |
|  |  |  | CV=0.26 |  |  |  |  |
| II c | CaWRKY03 | Capana01g002803 | 2 | 2 2 | 2 2（22/44）（50%） | 2  （7/44）  (15.9%) | 1 1  (6/44)（13.6%） |
|  | CaWRKY04 | Capana01g003441 | 3 | 0 2 2 |  |  |  |
|  | CaWRKY14 | Capana02g003661 | 1 | 2 |  |  |  |
|  | CaWRKY36 | Capana07g001968 | 2 | 2 2 |  |  |  |
|  | CaWRKY39 | Capana08g000429 | 2 | 1 2 |  |  |  |
|  | CaWRKY44 | Capana09g000676 | 2 | 2 2 |  |  |  |
|  | CaWRKY54 | Capana11g001905 | 2 | 2 2 |  |  |  |
|  | CaWRKY56 | Capana12g001826 | 2 | 2 2 |  |  |  |
|  | CaWRKY57 | Capana12g001851 | 2 | 2 2 |  |  |  |
|  | CaWRKY58 | Capana00g000429 | 1 | 2 |  |  |  |
|  | CaWRKY59 | Capana00g001033 | 1 | 2 |  |  |  |
|  | CaWRKY61 | Capana00g004112 | 2 | 2 2 |  |  |  |
|  | SlWRKY12 | solyc01g089960.2.1 | 3 | 0 2 2 |  |  |  |
|  | SlWRKY13 | solyc04g051540.2.1 | 2 | 2 2 |  |  |  |
|  | SlWRKY23 | solyc01g079260.2.1 | 2 | 2 2 |  |  |  |
|  | SlWRKY28 | solyc12g011200.1.1 | 2 | 2 2 |  |  |  |
|  | SlWRKY30 | solyc07g056280.2.1 | 2 | 2 2 |  |  |  |
|  | SlWRKY38 | solyc02g094270.1.1 | 1 | 2 |  |  |  |
|  | SlWRKY47 | solyc01g058540.2.1 | 2 | 2 2 |  |  |  |
|  | SlWRKY48 | solyc05g053380.2.1 | 2 | 2 2 |  |  |  |
|  | SlWRKY50 | solyc08g062490.2.1 | 2 | 1 2 |  |  |  |
|  | SlWRKY51 | solyc04g051690.2.1 | 2 | 0 2 |  |  |  |
|  | SlWRKY55 | solyc04g072070.2.1 | 2 | 2 2 |  |  |  |
|  | SlWRKY56 | solyc08g081630.1.1 | 1 | 2 |  |  |  |
|  | SlWRKY57 | solyc05g012500.2.1 | 2 | 2 2 |  |  |  |
|  | SlWRKY61 | solyc12g056750.1.1 | 2 | 2 2 |  |  |  |
|  | SlWRKY71 | solyc02g071130.2.1 | 2 | 2 2 |  |  |  |
|  | SlWRKY75 | solyc05g015850.2.1 | 1 | 2 |  |  |  |
|  | StWRKY15 | PGSC0003DMP400054315 | 3 | 0 22 |  |  |  |
|  | StWRKY16 | PGSC0003DMP400034248 | 2 | 1 1 |  |  |  |
|  | StWRKY23 | PGSC0003DMP400015928 | 2 | 2 2 |  |  |  |
|  | StWRKY24 | PGSC0003DMP400047290 | 2 | 1 1 |  |  |  |
|  | StWRKY33 | PGSC0003DMP400017131 | 2 | 2 2 |  |  |  |
|  | StWRKY34 | PGSC0003DMP400030318 | 2 | 1 1 |  |  |  |
|  | StWRKY35 | PGSC0003DMP400013709 | 2 | 2 2 |  |  |  |
|  | StWRKY59 | PGSC0003DMP400020289 | 2 | 1 1 |  |  |  |
|  | StWRKY60 | PGSC0003DMP400054257 | 2 | 2 2 |  |  |  |
|  | StWRKY61 | PGSC0003DMP400040153 | 2 | 1 1 |  |  |  |
|  | StWRKY62 | PGSC0003DMP400054355 | 2 | 2 2 |  |  |  |
|  | StWRKY63 | PGSC0003DMP400055959 | 1 | 1 |  |  |  |
|  | StWRKY68 | PGSC0003DMP400021797 | 1 | 1 |  |  |  |
|  | StWRKY69 | PGSC0003DMP400049367 | 2 | 1 1 |  |  |  |
|  | StWRKY80 | PGSC0003DMP400037922 | 1 | 2 |  |  |  |
|  | StWRKY81 | PGSC0003DMP400035081 | 3 | 0 2 2 |  |  |  |
|  |  |  | CV=0.27 |  |  |  |  |
| II d | CaWRKY08 | Capana02g000680 | 2 | 2 2 | 2 2（14/18）（77.8%） | 1 1  (2/14)  (14.3%) | 2 2 2  (1/14)  (7.1%) |
|  | CaWRKY22 | Capana03g003279 | 2 | 2 2 |  |  |  |
|  | CaWRKY23 | Capana04g000568 | 3 | 2 2 2 |  |  |  |
|  | CaWRKY30 | Capana06g003072 | 2 | 2 2 |  |  |  |
|  | CaWRKY60 | Capana00g003083 | 2 | 2 2 |  |  |  |
|  | SlWRKY07 | solyc04g078550.2.1 | 2 | 2 2 |  |  |  |
|  | SlWRKY08 | solyc02g093050.2.1 | 2 | 2 2 |  |  |  |
|  | SlWRKY10 | solyc12g096350.1.1 | 2 | 2 2 |  |  |  |
|  | SlWRKY11 | solyc08g006320.2.1 | 2 | 2 2 |  |  |  |
|  | SlWRKY21 | solyc06g008610.2.1 | 2 | 2 2 |  |  |  |
|  | SlWRKY24 | Solyc09g066010.2.1 | 0 |  |  |  |  |
|  | StWRKY09 | PGSC0003DMP400013975 | 2 | 2 2 |  |  |  |
|  | StWRKY10 | PGSC0003DMP400043322 | 2 | 2 2 |  |  |  |
|  | StWRKY11 | PGSC0003DMP400044188 | 2 | 2 2 |  |  |  |
|  | StWRKY13 | PGSC0003DMP400016838 | 2 | 1 1 |  |  |  |
|  | StWRKY14 | PGSC0003DMP400051153 | 2 | 1 1 |  |  |  |
|  | StWRKY18 | PGSC0003DMP400026478 | 2 | 2 2 |  |  |  |
|  | StWRKY19 | PGSC0003DMP400009437 | 2 | 2 2 |  |  |  |
|  |  |  | CV=0.28 |  |  |  |  |
| II e | CaWRKY02 | Capana01g000167 | 2 | 2 2 | 2 2 (16/21) (76.2%) | 1 1  (4/21) (19.0%) | 2 2 1  (1/21)（4.8%） |
|  | CaWRKY07 | Capana02g000212 | 2 | 2 2 |  |  |  |
|  | CaWRKY10 | Capana02g001642 | 2 | 2 2 |  |  |  |
|  | CaWRKY35 | Capana07g001809 | 2 | 2 2 |  |  |  |
|  | CaWRKY41 | Capana08g001012 | 2 | 2 2 |  |  |  |
|  | CaWRKY48 | Capana10g000754 | 2 | 2 2 |  |  |  |
|  | SlWRKY22 | solyc01g095100.2.1 | 2 | 2 2 |  |  |  |
|  | SlWRKY25 | solyc10g011910.2.1 | 2 | 2 2 |  |  |  |
|  | SlWRKY29 | solyc08g081610.2.1 | 2 | 2 2 |  |  |  |
|  | SlWRKY35 | solyc02g021680.2.1 | 2 | 2 2 |  |  |  |
|  | SlWRKY37 | solyc01g079360.2.1 | 2 | 2 2 |  |  |  |
|  | SlWRKY77 | solyc10g007970.1.1 | 2 | 2 2 |  |  |  |
|  | SlWRKY78 | solyc07g055280.2.1 | 2 | 2 2 |  |  |  |
|  | SlWRKY79 | solyc02g072190.2.1 | 2 | 2 2 |  |  |  |
|  | StWRKY20 | PGSC0003DMP400000156 | 2 | 2 2 |  |  |  |
|  | StWRKY36 | PGSC0003DMP400021798 | 2 | 1 1 |  |  |  |
|  | StWRKY46 | PGSC0003DMP400026520 | 2 | 2 2 |  |  |  |
|  | StWRKY47 | PGSC0003DMP400015849 | 2 | 1 1 |  |  |  |
|  | StWRKY70 | PGSC0003DMP400049560 | 2 | 1 1 |  |  |  |
|  | StWRKY71 | PGSC0003DMP400035491 | 3 | 2 2 1 |  |  |  |
|  | StWRKY72 | PGSC0003DMP400018674 | 2 | 1 1 |  |  |  |
|  |  |  | CV=0.11 |  |  |  |  |
| II f | CaWRKY17 | Capana03g001962 | 0 |  |  |  |  |
|  | SlWRKY26 | solyc03g082810.1.1 | 0 |  |  |  |  |
|  | StWRKY17 | PGSC0003DMP400066946 | 0 |  |  |  |  |
|  | StWRKY22 | PGSC0003DMP400067480 | 0 |  |  |  |  |
|  | StWRKY73 | PGSC0003DMP400056580 | 0 |  |  |  |  |
|  |  |  | CV=0 |  |  |  |  |
| II g | CaWRKY19 | Capana03g002134 | 2 | 2 2 | 2 2  (7/15)（46.7%） | 1 1  （6/15）（40%） | 1 2/1 1 2 0（1/15）（6.7%） |
|  | SlWRKY62 | solyc05g050040.1.1 | 2 | 2 2 |  |  |  |
|  | SlWRKY63 | solyc05g050050.1.1 | 2 | 2 2 |  |  |  |
|  | SlWRKY64 | solyc05g050060.1.1 | 2 | 2 2 |  |  |  |
|  | SlWRKY65 | solyc05g045710.1.1 | 2 | 1 2 |  |  |  |
|  | SlWRKY66 | solyc05g045880.1.1 | 2 | 2 2 |  |  |  |
|  | SlWRKY67 | solyc05g045800.1.1 | 2 | 1 1 |  |  |  |
|  | SlWRKY68 | solyc03g007640.1.1 | 2 | 2 2 |  |  |  |
|  | SlWRKY69 | solyc04g050210.1.1 | 2 | 2 2 |  |  |  |
|  | StWRKY25 | PGSC0003DMP400057959 | 2 | 1 1 |  |  |  |
|  | StWRKY26 | PGSC0003DMP400058743 | 2 | 1 1 |  |  |  |
|  | StWRKY27 | PGSC0003DMP400063301 | 2 | 1 1 |  |  |  |
|  | StWRKY28 | PGSC0003DMP400062598 | 4 | 1 1 2 0 |  |  |  |
|  | StWRKY29 | PGSC0003DMP400061279 | 2 | 1 1 |  |  |  |
|  | StWRKY30 | PGSC0003DMP400059804 | 1 | 1 1 |  |  |  |
|  |  |  | CV=0.29 |  |  |  |  |
| III | CaWRKY05 | Capana01g004471 | 3 | 0 2 2 | 2 2  （20/34）  （58.8%） | 1 1  （6/34）（17.6%） | 2 2 2/2  （2/34）（5.9%） |
|  | CaWRKY06 | Capana01g004472 | 1 | 2 |  |  |  |
|  | CaWRKY18 | Capana03g002072 | 2 | 2 2 |  |  |  |
|  | CaWRKY20 | Capana03g002635 | 2 | 2 2 |  |  |  |
|  | CaWRKY29 | Capana06g002128 | 2 | 2 2 |  |  |  |
|  | CaWRKY32 | Capana07g000528 | 2 | 1 2 |  |  |  |
|  | CaWRKY42 | Capana08g001044 | 2 | 2 2 |  |  |  |
|  | CaWRKY49 | Capana10g001220 | 1 | 2 |  |  |  |
|  | CaWRKY50 | Capana10g001548 | 2 | 2 2 |  |  |  |
|  | SlWRKY19 | solyc06g048870.1.1 | 3 | 2 2 2 |  |  |  |
|  | SlWRKY41 | solyc01g095630.2.1 | 2 | 2 2 |  |  |  |
|  | SlWRKY42 | solyc10g009550.2.1 | 2 | 2 2 |  |  |  |
|  | SlWRKY52 | solyc03g007380.1.1 | 2 | 2 2 |  |  |  |
|  | SlWRKY53 | solyc08g008280.2.1 | 2 | 2 2 |  |  |  |
|  | SlWRKY54 | solyc08g082110.2.1 | 2 | 2 2 |  |  |  |
|  | SlWRKY58 | solyc05g050340.2.1 | 2 | 2 2 |  |  |  |
|  | SlWRKY59 | solyc05g050330.2.1 | 2 | 2 2 |  |  |  |
|  | SlWRKY60 | solyc05g050300.1.1 | 1 | 1 |  |  |  |
|  | SlWRKY80 | solyc03g095770.2.1 | 2 | 2 2 |  |  |  |
|  | SlWRKY81 | solyc09g015770.2.1 | 2 | 2 2 |  |  |  |
|  | StWRKY37 | PGSC0003DMP400034520 | 2 | 2 2 |  |  |  |
|  | StWRKY53 | PGSC0003DMP400016026 | 2 | 2 2 |  |  |  |
|  | StWRKY54 | PGSC0003DMP400000453 | 2 | 1 1 |  |  |  |
|  | StWRKY55 | PGSC0003DMP400056273 | 2 | 2 2 |  |  |  |
|  | StWRKY56 | PGSC0003DMP400031375 | 2 | 1 1 |  |  |  |
|  | StWRKY57 | PGSC0003DMP400056261 | 2 | 1 1 |  |  |  |
|  | StWRKY64 | PGSC0003DMP400010348 | 2 | 2 2 |  |  |  |
|  | StWRKY65 | PGSC0003DMP400021486 | 2 | 2 2 |  |  |  |
|  | StWRKY66 | PGSC0003DMP400010350 | 2 | 0 0 |  |  |  |
|  | StWRKY67 | PGSC0003DMP400015423 | 3 | 2 2 2 |  |  |  |
|  | StWRKY74 | PGSC0003DMP400035817 | 2 | 1 1 |  |  |  |
|  | StWRKY75 | PGSC0003DMP400050864 | 2 | 2 2 |  |  |  |
|  | StWRKY76 | PGSC0003DMP400014725 | 2 | 1 1 |  |  |  |
|  | StWRKY77 | PGSC0003DMP400047973 | 2 | 1 1 |  |  |  |
|  |  |  | CV=0.21 |  |  |  |  |
| NG | CaWRKY01 | Capana01g003441 | 1 | 2 | 2 2  (3/11)  (27.3%) | 1 1/2  (2/11)（18.2%） | 0 0/1 0  (1/11)  （9.1%） |
|  | CaWRKY12 | Capana02g003053 | 2 | 2 2 |  |  |  |
|  | CaWRKY46 | Capana09g001790 | 2 | 2 2 |  |  |  |
|  | CaWRKY52 | Capana10g001805 | 2 | 0 0 |  |  |  |
|  | SlWRKY27 | solyc03g082750.1.1 | 0 |  |  |  |  |
|  | SlWRKY49 | solyc09g010960.2.1 | 2 | 2 2 |  |  |  |
|  | SlWRKY70 | solyc05g014040.1.1 | 1 | 2 |  |  |  |
|  | StWRKY21 | PGSC0003DMP400033713 | 2 | 1 1 |  |  |  |
|  | StWRKY31 | PGSC0003DMP400060373 | 2 | 1 0 |  |  |  |
|  | StWRKY32 | PGSC0003DMP400066732 | 1 | 1 |  |  |  |
|  | StWRKY45 | PGSC0003DMP400031479 | 0 |  |  |  |  |
|  |  |  | CV=0.59 |  |  |  |  |
