# Supplementary material for: Comparative Genomic Analysis Reveals Extensive Genetic Variations of WRKYs in Solanaceae and Functional Variations of CaWRKYs in Pepper
Source: Front Genet. 2019 May 28;10:492. doi: 10.3389/fgene.2019.00492 (PMC6546733; doi:10.3389/fgene.2019.00492)
Supplement: TABLE S2 — The group-wise analysis of WRKY domain frame, conserved amino acids number and zinc-finger structure. [file Table_2.DOCX]

| **Group** | **WRKY domain frame** | **NO. of conserved amino acids/**  **Zinc-finger structure** |
| --- | --- | --- |
| I-N | [DGYN]-WRKYGQK-X(1)-[VK]-X(5)-[RSYYK]-C-[T]-X(3)-C-X(2)-[KK]-X(7,8)-[G]-X(4)-[I]-X(1)-[Y]-X(3)-H-X(1)-H | 28; CX_4_CX_22-23_HXH |
| I-C | [DGY]-X(1)-WRKYGQK-X(1)-[V]-X(1)-[GN]-X(2)-[P]-X(0,6)-[RSYY]-X(1)-C-X(4)-C-X(1)-[V]-X(1)-[K]-X(1)-[VERA]-X(6)-[VITTYEG]-X(1)-H-X(1)-H | 35; CX_4_CX_23_HXH |
| II a | [DGY]-X(1)-WRKYGQK-[VTRDNP]-X(1)-[PRAY]-X(2)-C-[SFAP]-X(1)-C-  [PVKKKVQRS]-X(2)-[D]-X(1)-[SI]-X(1)-[VA]-X(1)-[YEGE]-H-[N]-H | 46; CX_4_CX_23_HXH |
| II b | [DGCQ]-WRKYGQK-X(3)-[G]-X(1)-[P]-X(0,1)-[CPRAYYR]-C-X(2)-[A]-X(2)-C-  [PVRKQVQRC]-X(2)-[D]-X(2)-[IL]-X(1)-[TTYEGT]-H-[N]-H | 43; CX_5_CX_23_HXH |
| II c | [DG]-X(2)-WRKYGQK-X(1)-[VK]-X(5,6)-[R]-X(1,7)-[YY]-X(1)-C-X(4)-C-X(1)-[VKK]-X(1)-[V]-X(1)-[R]-X(3)-[D]-X(5)-[TTYEG]-X(1)-H-X(1)-H | 29; CX_4_CX_23_HXH |
| II d | [D]-X(2)-S-WRKYGQK-[PIKGS]-X(2)-[PR]-X(1)-[YY]-X(1)-C-[SS]-X(1)-[RG]-C-  [PARKHVER]-X(6)-[ML]-X(1)-[VTY]-X(2)-[E]-H-X(1)-H | 40; CX_5_CX_23_HXH |
| II e | [D]-X(3)-WRKYGQK-[PIKGSPYPR]-X(1)-[YY]-X(1)-C-[S]-X(1)-[SKG]-C-X(1)-[A]-  X(1)-[KQVE]-X(11)-[TY]-X(3)-H-[N]-H | 35; CX_5_CX_23_HXH |
| II f | [QWR]-W-X(7)-[TG]-X(2,5)-[K]-X(1)-[YY]-X(1)-C-X(5,6)-C-[PA]-X(1)-[RH]-X(3)-  [SS]-X(1)-[D]-X(3)-[VIVTY]-X(1)-[G]-X(1)-H-X(1)-H | 26; CX_5-6_CX_23_HXH |
| II g | [D]-X(1)-[W]-X(1)-W-X(1)-K-X(1)-GQK-X(1)-[IK]-X(1)-[S]-X(1)-[FPRNY]-X(2)-C-  X(4,5)-C-X(1)-[A]-X(1)-[K]-X(1)-[IEKS]-X(3)-[E]-X(2)-[F]-X(1)-[VS]-X(1)-[S]-X(2)-H-[N]-H | 31; CX_4-5_CX_23_HXH |
| III | [DG]-X(2)-WRKYGQK-X(1)-[IL]-X(2,4)-[PR]-X(1)-[Y]-X(1)-[R]-C-X(4,7)-C-X(1)-[A]-X(1)-[K]-X(1)-[V]-X(11,12,13)-[TY]-X(3)-H-X(1)-C | 24; CX_4,7_CX_22-24_HXC |

Supplementary Table S2 The group-wise analysis of WRKY domain frame, conserved amino acids number and zinc-finger structure

**Note:** The amino acids with occurrence rate over 90% were labeled in []; “X” indicate the random amino acids; The symbolic heptapeptide WRKYGQK and C_2_H_2_ of zinc-finger structure were underlined.
