# Supplementary material for: Comparative Genomic Analysis Reveals Extensive Genetic Variations of WRKYs in Solanaceae and Functional Variations of CaWRKYs in Pepper
Source: Front Genet. 2019 May 28;10:492. doi: 10.3389/fgene.2019.00492 (PMC6546733; doi:10.3389/fgene.2019.00492)
Supplement: TABLE S3 — Domain composition analysis in WRKY proteins of solanaceous plants. [file Table_3.DOCX]

Supplementary Table S3 Domain composition analysis in WRKY proteins of solanaceous plants

|  | **Group** | **Gene symbol** | **Protein ID** | **Plant WRKY domain** | | **Domain organization** | |
| --- | --- | --- | --- | --- | --- | --- | --- |
|  |  |  |  | **Yes/No** | **Length** |  |  |
| Pepper (*Capsicum annuum L.*) | I | CaWRKY13 | Capana02g003339 | Yes | 56/59 | 2WRKY |  |
|  |  | CaWRKY21 | Capana03g003085 | Yes | 58/59 | 2WRKY |  |
|  |  | CaWRKY24 | Capana04g001820 | Yes | 57/59 | 2WRKY |  |
|  |  | CaWRKY25 | Capana05g002502 | Yes | 58/58 | 2WRKY |  |
|  |  | CaWRKY28 | Capana06g001506 | Yes | 58/59 | 2WRKY |  |
|  |  | CaWRKY31 | Capana07g000181 | Yes | 57/58 | 2WRKY |  |
|  |  | CaWRKY33 | Capana07g001256 | Yes | 58/59 | 2WRKY, WD40 |  |
|  |  | CaWRKY37 | Capana07g002350 | Yes | 59/59 | 2WRKY |  |
|  |  | CaWRKY38 | Capana07g002454 | Yes | 59/59 | 2WRKY |  |
|  |  | CaWRKY45 | Capana09g001251 | Yes | 59/59 | 2WRKY |  |
|  |  | CaWRKY47 | Capana10g000205 | Yes* | 59/40 | 2WRKY |  |
|  |  | CaWRKY51 | Capana10g001791 | Yes* | 57/57 | 2WRKY |  |
|  |  | CaWRKY53 | Capana11g001882 | Yes | 57/59 | 2WRKY |  |
|  | II a | CaWRKY15 | Capana03g000473 | Yes | 61 | WRKY |  |
|  |  | CaWRKY27 | Capana06g001110 | Yes | 60 | WRKY |  |
|  |  | CaWRKY40 | Capana08g000683 | Yes | 60 | WRKY |  |
|  |  | CaWRKY55 | Capana12g001134 | Yes | 60 | WRKY |  |
|  | II b | CaWRKY09 | Capana02g000918 | Yes | 59 | WRKY |  |
|  |  | CaWRKY11 | Capana02g002230 | Yes | 59 | WRKY |  |
|  |  | CaWRKY16 | Capana03g001099 | Yes | 60 | WRKY |  |
|  |  | CaWRKY26 | Capana06g001008 | Yes | 60 | WRKY |  |
|  |  | CaWRKY34 | Capana07g001387 | Yes | 59 | WRKY |  |
|  |  | CaWRKY43 | Capana08g001961 | Yes | 61 | WRKY |  |
|  | II c | CaWRKY03 | Capana01g002803 | Yes | 54 | WRKY |  |
|  |  | CaWRKY04 | Capana01g003441 | Yes | 59 | WRKY |  |
|  |  | CaWRKY14 | Capana02g003661 | Yes | 59 | WRKY |  |
|  |  | CaWRKY36 | Capana07g001968 | Yes | 58 | WRKY |  |
|  |  | CaWRKY39 | Capana08g000429 | Yes* | 59 | WRKY |  |
|  |  | CaWRKY44 | Capana09g000676 | Yes | 58 | WRKY |  |
|  |  | CaWRKY54 | Capana11g001905 | Yes | 58 | WRKY |  |
|  |  | CaWRKY56 | Capana12g001826 | Yes* | 59 | WRKY |  |
|  |  | CaWRKY57 | Capana12g001851 | Yes | 59 | WRKY |  |
|  |  | CaWRKY58 | Capana00g000429 | Yes* | 49 | WRKY |  |
|  |  | CaWRKY59 | Capana00g001033 | Yes | 59 | WRKY |  |
|  |  | CaWRKY61 | Capana00g004112 | Yes* | 59 | WRKY |  |
|  | II d | CaWRKY08 | Capana02g000680 | Yes | 58 | WRKY, PIANT-ZN-CIUST |  |
|  |  | CaWRKY22 | Capana03g003279 | Yes | 58 | WRKY, PIANT-ZN-CIUST |  |
|  |  | CaWRKY23 | Capana04g000568 |  | 58 | WRKY, PIANT-ZN-CIUST,  PTZ00112 |  |
|  |  | CaWRKY30 | Capana06g003072 | Yes | 59 | WRKY, PIANT-ZN-CIUST |  |
|  |  | CaWRKY60 | Capana00g003083 | Yes | 59 | WRKY |  |
|  | II e | CaWRKY02 | Capana01g000167 | Yes | 59 | WRKY |  |
|  |  | CaWRKY07 | Capana02g000212 | Yes | 59 | WRKY |  |
|  |  | CaWRKY10 | Capana02g001642 | Yes | 59 | WRKY |  |
|  |  | CaWRKY35 | Capana07g001809 | Yes | 58 | WRKY |  |
|  |  | CaWRKY41 | Capana08g001012 | Yes | 59 | WRKY |  |
|  |  | CaWRKY48 | Capana10g000754 | Yes | 59 | WRKY |  |
|  | II f | CaWRKY17 | Capana03g001962 | Yes* | 59 | WRKY |  |
|  | II g | CaWRKY19 | Capana03g002134 | Yes | 59 | WRKY |  |
|  | III | CaWRKY05 | Capana01g004471 | Yes | 61 | WRKY |  |
|  |  | CaWRKY06 | Capana01g004472 | Yes | 61 | WRKY |  |
|  |  | CaWRKY18 | Capana03g002072 | Yes | 62 | WRKY |  |
|  |  | CaWRKY20 | Capana03g002635 | Yes | 63 | WRKY |  |
|  |  | CaWRKY29 | Capana06g002128 | Yes | 60 | WRKY |  |
|  |  | CaWRKY32 | Capana07g000528 | Yes | 59 | WRKY |  |
|  |  | CaWRKY42 | Capana08g001044 | Yes | 61 | WRKY |  |
|  |  | CaWRKY49 | Capana10g001220 | Yes | 62 | WRKY |  |
|  |  | CaWRKY50 | Capana10g001548 | Yes | 62 | WRKY |  |
|  | NG | CaWRKY01 | Capana01g000165 | Yes | 23 | WRKY |  |
|  |  | CaWRKY12 | Capana02g003053 | Yes | 19 | WRKY, PIANT-ZN-CIUST |  |
|  |  | CaWRKY46 | Capana09g001790 | Yes | 57 | WRKY |  |
|  |  | CaWRKY52 | Capana10g001805 | Yes | 57 | WRKY |  |
| Tomato (*Solanum lycopersicum*) | I | SlWRKY01 | solyc07g047960.2.1 | Yes | 65/66 | 2WRKY |  |
|  |  | SlWRKY02 | solyc07g066220.2.1 | Yes | 65/66 | 2WRKY |  |
|  |  | SlWRKY03 | solyc02g088340.2.1 | Yes | 65/66 | 2WRKY |  |
|  |  | SlWRKY04 | solyc05g012770.2.1 | Yes | 65/66 | 2WRKY |  |
|  |  | SlWRKY05 | solyc03g104810.2.1 | Yes | 65/66 | 2WRKY |  |
|  |  | SlWRKY14 | solyc12g006170.1.1 | Yes | 65/66 | 2WRKY |  |
|  |  | SlWRKY15 | solyc10g005680.1.1 | Yes* | 65/65 | 2WRKY |  |
|  |  | SlWRKY18 | solyc07g065260.2.1 | Yes | 65/66 | 2WRKY |  |
|  |  | SlWRKY20 | solyc12g014610.1.1 | Yes | 65/66 | 2WRKY |  |
|  |  | SlWRKY31 | solyc06g066370.2.1 | Yes | 65/66 | 2WRKY |  |
|  |  | SlWRKY32 | solyc07g005650.2.1 | Yes | 65/66 | 2WRKY |  |
|  |  | SlWRKY33 | solyc09g014990.2.1 | Yes | 65/66 | 2WRKY |  |
|  |  | SlWRKY34 | solyc05g055750.2.1 | Yes | 65/66 | 2WRKY |  |
|  |  | SlWRKY36 | solyc04g056360.2.1 | Yes | 65/66 | 2WRKY |  |
|  |  | SlWRKY44 | solyc10g084380.1.1 | Yes | 65/66 | 2WRKY |  |
|  | II a | SlWRKY39 | solyc03g116890.2.1 | Yes | 67 | WRKY |  |
|  |  | SlWRKY40 | solyc06g068460.2.1 | Yes | 67 | WRKY, MreC, PHA03255, SSP160 |  |
|  |  | SlWRKY43 | solyc12g042590.1.1 | Yes | 67 | WRKY |  |
|  |  | SlWRKY45 | solyc08g067360.2.1 | Yes | 67 | WRKY |  |
|  |  | SlWRKY46 | solyc08g067340.2.1 | Yes | 67 | WRKY, FPP |  |
|  | II b | SlWRKY06 | solyc02g080890.2.1 | Yes | 67 | WRKY, bZIP Maf_small,  ZapB |  |
|  |  | SlWRKY09 | solyc01g104550.2.1 | Yes | 68 | WRKY |  |
|  |  | SlWRKY16 | solyc02g032950.2.1 | Yes | 67 | WRKY, Mrec, ATG16  DNA bind RsfA, COG4372 |  |
|  |  | SlWRKY17 | solyc07g051840.2.1 | Yes | 67 | WRKY, bZIP Maf small |  |
|  |  | SlWRKY72 | solyc02g067430.2.1 | Yes | 67 | WRKY |  |
|  |  | SlWRKY73 | solyc03g113120.2.1 | Yes | 67 | WRKY |  |
|  |  | SlWRKY74 | solyc06g070990.2.1 | Yes | 67 | WRKY |  |
|  |  | SlWRKY76 | solyc05g007110.2.1 | Yes | 67 | WRKY |  |
|  | II c | SlWRKY12 | solyc01g089960.2.1 | Yes | 66 | WRKY |  |
|  |  | SlWRKY13 | solyc04g051540.2.1 | Yes | 66 | WRKY |  |
|  |  | SlWRKY23 | solyc01g079260.2.1 | Yes | 66 | WRKY |  |
|  |  | SlWRKY28 | solyc12g011200.1.1 | Yes | 66 | WRKY |  |
|  |  | SlWRKY30 | solyc07g056280.2.1 | Yes | 66 | WRKY |  |
|  |  | SlWRKY38 | solyc02g094270.1.1 | Yes | 66 | WRKY |  |
|  |  | SlWRKY47 | solyc01g058540.2.1 | Yes | 66 | WRKY |  |
|  |  | SlWRKY48 | solyc05g053380.2.1 | Yes | 66 | WRKY |  |
|  |  | SlWRKY50 | solyc08g062490.2.1 | Yes* | 66 | WRKY |  |
|  |  | SlWRKY51 | solyc04g051690.2.1 | Yes | 66 | WRKY |  |
|  |  | SlWRKY55 | solyc04g072070.2.1 | Yes* | 66 | WRKY |  |
|  |  | SlWRKY56 | solyc08g081630.1.1 | Yes | 66 | WRKY |  |
|  |  | SlWRKY57 | solyc05g012500.2.1 | Yes | 66 | WRKY |  |
|  |  | SlWRKY61 | solyc12g056750.1.1 | Yes* | 67 | WRKY |  |
|  |  | SlWRKY71 | solyc02g071130.2.1 | Yes | 66 | WRKY |  |
|  |  | SlWRKY75 | solyc05g015850.2.1 | Yes | 66 | WRKY |  |
|  | II d | SlWRKY07 | solyc04g078550.2.1 | Yes | 67 | WRKY, PIANT-ZN-CIUST |  |
|  |  | SlWRKY08 | solyc02g093050.2.1 | Yes | 67 | WRKY, PIANT-ZN-CIUST |  |
|  |  | SlWRKY10 | solyc12g096350.1.1 | Yes | 67 | WRKY, PIANT-ZN-CIUST |  |
|  |  | SlWRKY11 | solyc08g006320.2.1 | Yes | 67 | WRKY, PIANT-ZN-CIUST |  |
|  |  | SlWRKY21 | solyc06g008610.2.1 | Yes | 67 | WRKY, PIANT-ZN-CIUST |  |
|  |  | SlWRKY24 | Solyc09g066010.2.1 | Yes | 67 | WRKY, PIANT-ZN-CIUST |  |
|  | II e | SlWRKY22 | solyc01g095100.2.1 | Yes | 67 | WRKY |  |
|  |  | SlWRKY25 | solyc10g011910.2.1 | Yes | 67 | WRKY |  |
|  |  | SlWRKY29 | solyc08g081610.2.1 | Yes | 67 | WRKY |  |
|  |  | SlWRKY35 | solyc02g021680.2.1 | Yes | 67 | WRKY |  |
|  |  | SlWRKY37 | solyc01g079360.2.1 | Yes | 67 | WRKY |  |
|  |  | SlWRKY77 | solyc10g007970.1.1 | Yes | 67 | WRKY |  |
|  |  | SlWRKY78 | solyc07g055280.2.1 | Yes | 67 | WRKY |  |
|  |  | SlWRKY79 | solyc02g072190.2.1 | Yes | 67 | WRKY |  |
|  | II f | SlWRKY26 | solyc03g082810.1.1 | Yes | 67 | WRKY |  |
|  | II g | SlWRKY62 | solyc05g050040.1.1 | Yes* | 67 | WRKY |  |
|  |  | SlWRKY63 | solyc05g050050.1.1 | Yes | 67 | WRKY |  |
|  |  | SlWRKY64 | solyc05g050060.1.1 | Yes | 67 | WRKY |  |
|  |  | SlWRKY65 | solyc05g045710.1.1 | Yes | 67 | WRKY |  |
|  |  | SlWRKY66 | solyc05g045880.1.1 | Yes | 67 | WRKY |  |
|  |  | SlWRKY67 | solyc05g045800.1.1 | Yes | 67 | WRKY |  |
|  |  | SlWRKY68 | solyc03g007640.1.1 | Yes* | 66 | WRKY |  |
|  |  | SlWRKY69 | solyc04g050210.1.1 | Yes | 67 | WRKY |  |
|  | III | SlWRKY19 | solyc06g048870.1.1 | Yes | 65 | WRKY |  |
|  |  | SlWRKY41 | solyc01g095630.2.1 | Yes | 69 | WRKY |  |
|  |  | SlWRKY42 | solyc10g009550.2.1 | Yes | 69 | WRKY |  |
|  |  | SlWRKY52 | solyc03g007380.1.1 | Yes | 69 | WRKY |  |
|  |  | SlWRKY53 | solyc08g008280.2.1 | Yes | 69 | WRKY |  |
|  |  | SlWRKY54 | solyc08g082110.2.1 | Yes | 69 | WRKY |  |
|  |  | SlWRKY58 | solyc05g050340.2.1 | Yes | 66 | WRKY |  |
|  |  | SlWRKY59 | solyc05g050330.2.1 | Yes | 66 | WRKY |  |
|  |  | SlWRKY60 | solyc05g050300.1.1 | Yes* | 63 | WRKY |  |
|  |  | SlWRKY80 | solyc03g095770.2.1 | Yes | 70 | WRKY |  |
|  |  | SlWRKY81 | solyc09g015770.2.1 | Yes | 69 | WRKY |  |
|  | NG | SlWRKY27 | solyc03g082750.1.1 | Yes* | 45 | WRKY |  |
|  |  | SlWRKY49 | solyc09g010960.2.1 | Yes | 66 | WRKY |  |
|  |  | SlWRKY70 | solyc05g014040.1.1 | Yes | 41 | WRKY |  |
| Potato (*Solanum tuberosum*) | I | StWRKY01 | PGSC0003DMP400038283 | Yes | 59/60 | 2WRKY |  |
|  |  | StWRKY02 | PGSC0003DMP400019959 | Yes* | 59/60 | 2WRKY |  |
|  |  | StWRKY03 | PGSC0003DMP400049274 | Yes | 59/60 | 2WRKY, TMEM171 |  |
|  |  | StWRKY04 | PGSC0003DMP400009822 | Yes | 60/66 | 2WRKY |  |
|  |  | StWRKY05 | PGSC0003DMP400002598 | Yes | 59/60 | 2WRKY |  |
|  |  | StWRKY38 | PGSC0003DMP400029302 | Yes | 59/60 | 2WRKY |  |
|  |  | StWRKY39 | PGSC0003DMP400020631 | Yes | 59/35 | 2WRKY |  |
|  |  | StWRKY40 | PGSC0003DMP400051947 | Yes | 59/60 | 2WRKY |  |
|  |  | StWRKY41 | PGSC0003DMP400038615 | Yes | 59/60 | 2WRKY |  |
|  |  | StWRKY42 | PGSC0003DMP400040389 | Yes | 59/60 | 2WRKY, BCNT |  |
|  |  | StWRKY43 | PGSC0003DMP400050209 | Yes | 59/60 | 2WRKY |  |
|  |  | StWRKY44 | PGSC0003DMP400010920 | Yes | 59/60 | 2WRKY |  |
|  |  | StWRKY58 | PGSC0003DMP400019408 | Yes | 59/60 | 2WRKY |  |
|  | II a | StWRKY48 | PGSC0003DMP400049656 | Yes | 61 | WRKY, PHA03255,SSP160 |  |
|  |  | StWRKY49 | PGSC0003DMP400034429 | Yes | 61 | WRKY |  |
|  |  | StWRKY50 | PGSC0003DMP400013078 | Yes | 61 | WRKY |  |
|  |  | StWRKY51 | PGSC0003DMP400013081 | Yes | 61 | WRKY, DNA_bind_RsfA |  |
|  |  | StWRKY52 | PGSC0003DMP400049886 | Yes | 61 | WRKY |  |
|  | II b | StWRKY06 | PGSC0003DMP400028763 | Yes | 61 | WRKY, bZIP Maf small |  |
|  |  | StWRKY07 | PGSC0003DMP400026405 | Yes | 61 | WRKY, bZIP Maf small |  |
|  |  | StWRKY08 | PGSC0003DMP400038147 | Yes | 61 | WRKY |  |
|  |  | StWRKY12 | PGSC0003DMP400051886 | Yes | 62 | WRKY |  |
|  |  | StWRKY78 | PGSC0003DMP400012281 | Yes | 61 | WRKY |  |
|  |  | StWRKY79 | PGSC0003DMP400031503 | Yes | 61 | WRKY, PRKG1 interact,  PRK03918 |  |
|  | II c | StWRKY15 | PGSC0003DMP400054315 | Yes | 60 | WRKY |  |
|  |  | StWRKY16 | PGSC0003DMP400034248 | Yes | 60 | WRKY |  |
|  |  | StWRKY23 | PGSC0003DMP400015928 | Yes | 60 | WRKY |  |
|  |  | StWRKY24 | PGSC0003DMP400047290 | Yes | 60 | WRKY |  |
|  |  | StWRKY33 | PGSC0003DMP400017131 | Yes | 60 | WRKY |  |
|  |  | StWRKY34 | PGSC0003DMP400030318 | Yes | 60 | WRKY |  |
|  |  | StWRKY35 | PGSC0003DMP400013709 | Yes | 60 | WRKY |  |
|  |  | StWRKY59 | PGSC0003DMP400020289 | Yes | 60 | WRKY |  |
|  |  | StWRKY60 | PGSC0003DMP400054257 | Yes* | 60 | WRKY |  |
|  |  | StWRKY61 | PGSC0003DMP400040153 | Yes* | 61 | WRKY |  |
|  |  | StWRKY62 | PGSC0003DMP400054355 | Yes* | 66 | WRKY, MraZ N |  |
|  |  | StWRKY63 | PGSC0003DMP400055959 | Yes* | 61 | WRKY |  |
|  |  | StWRKY68 | PGSC0003DMP400021797 | Yes | 60 | WRKY |  |
|  |  | StWRKY69 | PGSC0003DMP400049367 | Yes | 60 | WRKY |  |
|  |  | StWRKY80 | PGSC0003DMP400037922 | Yes | 60 | WRKY |  |
|  |  | StWRKY81 | PGSC0003DMP400035081 | Yes | 60 | WRKY |  |
|  | II d | StWRKY09 | PGSC0003DMP400013975 | Yes | 61 | WRKY，PIANT-ZN-CIUST |  |
|  |  | StWRKY10 | PGSC0003DMP400043322 | Yes | 61 | WRKY，PIANT-ZN-CIUST |  |
|  |  | StWRKY11 | PGSC0003DMP400044188 | Yes | 61 | WRKY，PIANT-ZN-CIUST |  |
|  |  | StWRKY13 | PGSC0003DMP400016838 | Yes | 61 | WRKY，PIANT-ZN-CIUST |  |
|  |  | StWRKY14 | PGSC0003DMP400051153 | Yes | 61 | WRKY，PIANT-ZN-CIUST |  |
|  |  | StWRKY18 | PGSC0003DMP400026478 | Yes | 61 | WRKY, PIANT-ZN-CIUST |  |
|  |  | StWRKY19 | PGSC0003DMP400009437 | Yes | 61 | WRKY, PIANT-ZN-CIUST |  |
|  | II e | StWRKY20 | PGSC0003DMP400000156 | Yes | 61 | WRKY |  |
|  |  | StWRKY36 | PGSC0003DMP400021798 | Yes | 61 | WRKY |  |
|  |  | StWRKY46 | PGSC0003DMP400026520 | Yes | 61 | WRKY |  |
|  |  | StWRKY47 | PGSC0003DMP400015849 | Yes | 61 | WRKY |  |
|  |  | StWRKY70 | PGSC0003DMP400049560 | Yes | 61 | WRKY |  |
|  |  | StWRKY71 | PGSC0003DMP400035491 | Yes | 61 | WRKY |  |
|  |  | StWRKY72 | PGSC0003DMP400018674 | Yes | 61 | WRKY |  |
|  | II f | StWRKY17 | PGSC0003DMP400066946 | Yes* | 61 | WRKY |  |
|  |  | StWRKY22 | PGSC0003DMP400067480 | Yes* | 57 | WRKY |  |
|  |  | StWRKY73 | PGSC0003DMP400056580 | Yes* | 56 | WRKY |  |
|  | II g | StWRKY25 | PGSC0003DMP400057959 | Yes | 61 | WRKY |  |
|  |  | StWRKY26 | PGSC0003DMP400058743 | Yes | 61 | WRKY |  |
|  |  | StWRKY27 | PGSC0003DMP400063301 | Yes | 61 | WRKY |  |
|  |  | StWRKY28 | PGSC0003DMP400062598 | Yes | 61 | WRKY |  |
|  |  | StWRKY29 | PGSC0003DMP400061279 | Yes* | 61 | WRKY |  |
|  |  | StWRKY30 | PGSC0003DMP400059804 | Yes* | 55 | WRKY |  |
|  | III | StWRKY37 | PGSC0003DMP400034520 | Yes | 63 | WRKY |  |
|  |  | StWRKY53 | PGSC0003DMP400016026 | Yes | 63 | WRKY |  |
|  |  | StWRKY54 | PGSC0003DMP400000453 | Yes | 63 | WRKY |  |
|  |  | StWRKY55 | PGSC0003DMP400056273 | Yes* | 60 | WRKY |  |
|  |  | StWRKY56 | PGSC0003DMP400031375 | Yes | 64 | WRKY |  |
|  |  | StWRKY57 | PGSC0003DMP400056261 | Yes | 60 | WRKY |  |
|  |  | StWRKY64 | PGSC0003DMP400010348 | Yes | 63 | WRKY |  |
|  |  | StWRKY65 | PGSC0003DMP400021486 | Yes | 63 | WRKY |  |
|  |  | StWRKY66 | PGSC0003DMP400010350 | Yes | 54 | WRKY |  |
|  |  | StWRKY67 | PGSC0003DMP400015423 | Yes | 63 | WRKY |  |
|  |  | StWRKY74 | PGSC0003DMP400035817 | Yes | 64 | WRKY |  |
|  |  | StWRKY75 | PGSC0003DMP400050864 | Yes | 63 | WRKY |  |
|  |  | StWRKY76 | PGSC0003DMP400014725 | Yes | 63 | WRKY |  |
|  |  | StWRKY77 | PGSC0003DMP400047973 | Yes | 64 | WRKY |  |
|  | NG | StWRKY21 | PGSC0003DMP400033713 | Yes | 35 | WRKY |  |
|  |  | StWRKY31 | PGSC0003DMP400060373 | Yes* | 52 | WRKY |  |
|  |  | StWRKY32 | PGSC0003DMP400066732 | Yes* | 49 | WRKY |  |
|  |  | StWRKY45 | PGSC0003DMP400031479 | Yes* | 59 | WRKY |  |

**Abbreviation used in the table:** Plant-ZN-Clust (Plant zinc cluster domain), PTZ00112 (Origin recognition complex 1 protein), MreC (Murein formation C), PHA03225 (BDLF3), FPP (Filament-like plant protein), bZIP_Maf_small ( Basic leucine zipper (bZIP) domain of small musculoaponeurotic fibrosarcoma (Maf) proteins), ZapB (Cell division protein ZapB), ATG16 ( Autophagy protein 16 ), DNA_bind_RsfA (Transcription factor, RsfA family), COG4372 (Uncharacterized conserved protein, contains DUF3084 domain), TMEM171 (Transmembrane protein family 171), BCNT (Bucentaur or craniofacial development protein 1), SSP160 (Special lobe-specific silk protein 160), PRKG1 interact (cGMP-dependent protein kinase interacting domain), PRK03918 (Chromosome segregation protein), MraZ N(N-terminal subdomain of transcriptional regulator MraZ) The asterisks showed in 28 Solanaceae plant CNGCs as Yes* indicated variant WRKY domains. PRK13922,
