# Supplementary material for: Comparative Genomic Analysis Reveals Extensive Genetic Variations of WRKYs in Solanaceae and Functional Variations of CaWRKYs in Pepper
Source: Front Genet. 2019 May 28;10:492. doi: 10.3389/fgene.2019.00492 (PMC6546733; doi:10.3389/fgene.2019.00492)
Supplement: TABLE S4 — Primers of CaWRKY genes for RT-qPCR analysis. [file Table_4.DOCX]

Supplemental Table S4 Primers of *CaWRKY* genes for qRT-PCR analysis

| **Name of primer** | **Sequence (5'→ 3')** | **Anticipated PCR product (bp)** |
| --- | --- | --- |
| CaWRKY01-F | ATGGAAGGTGGTGAACAA |  |
| CaWRKY01-R | CCCTGCATAGCCTAGTTC | 255 |
| CaWRKY02-F | GAACAACTTTACAAGCCATT |  |
| CaWRKY02-R | CAACTGAAGCACCACTCT | 198 |
| CaWRKY03-F | AGAGAGCCGAGATTTGCATT |  |
| CaWRKY03-R | CCACTATGCTTGGGTCACTG | 190 |
| CaWRKY04-F | TAGGGTTTAGCCACAATG |  |
| CaWRKY04-R | TAACTCCTGGGATGAAGA | 298 |
| CaWRKY05-F | TCTTTCAACTGGCAATAC |  |
| CaWRKY05-R | ATATCCATCATCGGTAGG | 297 |
| CaWRKY06-F | AGTGCAGAGGTCAGACGATG |  |
| CaWRKY06-R | GAAATGACATGCTGCTTGCT | 241 |
| CaWRKY07-F | TCTCAATACCAAGGCGATAC |  |
| CaWRKY07-R | GCTTCAAGTTCCACCAAA | 257 |
| CaWRKY08-F | AAGTTGTCAGACATCCCACC |  |
| CaWRKY08-R | GGTTATGCTCGCTTTCGT | 190 |
| CaWRKY09-F | AGTTTCTCAGCCTAATGTC |  |
| CaWRKY09-R | CAATGGGAAGTGAGTGAT | 254 |
| CaWRKY10-F | ATTTGGATGGTTTTCTGA |  |
| CaWRKY10-R | GGTGTTAAGCTATGTCGG | 228 |
| CaWRKY11-F | AATGCTGCACTGCCTAAT |  |
| CaWRKY11-R | CCTGAGAAACTTGTAAACCC | 187 |
| CaWRKY12-F | TGCCATTGTTCCAAGAGAAG |  |
| CaWRKY12-R | TTTCATGAGGGCAAACAAAG | 176 |
| CaWRKY13-F | TTGAGAAATCCGTTGATG |  |
| CaWRKY13-R | ATGAGAAGGAGAAGACCC | 188 |
| CaWRKY14-F | GATGGCTATCGTTGGAGA |  |
| CaWRKY14-R | ATTATCATTGGGCTTGTC | 192 |
| CaWRKY15-F | CTAGCCGATTAAACCCGAAA |  |
| CaWRKY15-R | CATGTCCTCCTGGTGATTTG | 216 |
| CaWRKY16-F | CAGGGATGAAGAAGATGA |  |
| **Name of primer** | **Sequence (5'-3')** | **Anticipatd PCR product(bp)** |
| CaWRKY16-R | GTGGCTGAAAGAGGAAGT | 282 |
| CaWRKY17-F | GATGGAGAAAGTATGGGATG |  |
| CaWRKY17-R | TGTTGGTTAGGAGGAGGG | 178 |
| CaWRKY18-F | CATCTCAATGCTCCTAAC |  |
| CaWRKY18-R | ATCGTGGAGAATCAGACA | 202 |
| CaWRKY19-F | TAGGCTCCGAATCATTAA |  |
| CaWRKY19-R | AGAAACAGGAGTTGTGGG | 173 |
| CaWRKY20-F | ACGTTGGTTGATTCCAACAG |  |
| CaWRKY20-R | TGCACAACCATCATTCATTG | 213 |
| CaWRKY21-F | TGGCCAGAAACTTGTCAAAG |  |
| CaWRKY21-R | GGGTTTAGGATGATCATGGG | 151 |
| CaWRKY22-F | GGAAGCACAGGGTGAAGA |  |
| CaWRKY22-R | ATTTGCCGATTGAGATGG | 266 |
| CaWRKY23-F | TGCCACTGCCCTAAGAAA |  |
| CaWRKY23-R | GTAATGGAATGGGAATGA | 272 |
| CaWRKY24-F | ATGAGTTTAATGGTGGCT |  |
| CaWRKY24-R | AAGACTGGGATAATGCTG | 160 |
| CaWRKY25-F | GACGGATTTCCTGACGAT |  |
| CaWRKY25-R | TTGTTGCTGAGGGATTTG | 151 |
| CaWRKY26-F | TGCGAGAAGTAATGGAAG |  |
| CaWRKY26-R | TAGGCTAAGGGAAACAAG | 191 |
| CaWRKY27-F | GTATCTCCAATCCCAACA |  |
| CaWRKY27-R | CTGACCATACTTCCTCCA | 236 |
| CaWRKY28-F | CCGTGACATCTCATCAGTCC |  |
| CaWRKY28-R | CAGCAAAGCAATGACTCCAT | 235 |
| CaWRKY29-F | AGAATGGGAAATCTTGAGC |  |
| CaWRKY29-R | TGGAGGAGTTGTGGTGGT | 285 |
| CaWRKY30-F | GAATAATGCTGCCGTTGAGA |  |
| CaWRKY30-R | TTCCTCACTCTTGCATGACC | 171 |
| CaWRKY31-F | AAACCTGGAAAGAAACCC |  |
| **Name of primer** | **Sequence (5'→ 3')** | **Anticipated PCR product (bp)** |
| CaWRKY31-R | GTGCTATCTACAGCCCTC | 188 |
| CaWRKY32-F | ACAGCGTGATGAGGTTGG |  |
| CaWRKY32-R | ATCCGTGTCTGGATAAGGTGAA | 193 |
| CaWRKY33-F | TATGGCGTCCCTATTTAC |  |
| CaWRKY33-R | ATACCGCTATTTCCTTCTC | 170 |
| CaWRKY34-F | CGGAAGCAAGTTCAAAGA |  |
| CaWRKY34-R | TAGGTCCAATGTAACAGTAGG | 264 |
| CaWRKY35-F | CTTGGTCTTGGAGGAAATA |  |
| CaWRKY35-R | GTGGATGGTGAAGTAGCG | 235 |
| CaWRKY36-F | AAAGGCAGTGAAGAATAG |  |
| CaWRKY36-R | GAGGAGGATAAGAATGAA | 210 |
| CaWRKY37-F | CAAGAAGATAAATGCGAGAA |  |
| CaWRKY37-R | GTTACATCCATCCCACCA | 239 |
| CaWRKY38-F | ACTCGCTTCAATCTCACC |  |
| CaWRKY38-R | AACTTGTTTCTGCCCGTA | 290 |
| CaWRKY39-F | CAGCAGTTACCAGGAAGA |  |
| CaWRKY39-R | GGTTATCACATACCGAGA | 256 |
| CaWRKY40-F | ACCAAAGAGGCCAAGAGAAA |  |
| CaWRKY40-R | CATGTTGGTGCAAATGAACA | 169 |
| CaWRKY41-F | TCAAAGGGTTGTTTAGCC |  |
| CaWRKY41-R | AAATTCTTCGTCCTCGTC | 297 |
| CaWRKY42-F | AACACAAGGGAAGGAGTTCG |  |
| CaWRKY42-R | CCACCTTCAAGAAGAAGTGCT | 153 |
| CaWRKY43-F | TTAGGGTTATCTTTGACTTTGG |  |
| CaWRKY43-R | AGCCGATTCACATCTTGC | 234 |
| CaWRKY44-F | CCCAAATGCTAATTCCAC |  |
| CaWRKY44-R | ATGGCTCTTGTTTCTGGAT | 165 |
| CaWRKY45-F | GCACATTTATTGGCTGTC |  |
| CaWRKY45-R | CAAGGTGATTGGTGGTTC | 272 |
| CaWRKY46-F | AGAATAGCCCATATCCCA |  |
| CaWRKY46-R | CATCTTCAAGGTCCACAA | 294 |
| CaWRKY47-F | TCCTGCCGAAGATGGTTA |  |
| CaWRKY47-R | TGGCTTAGGGTGATTGTG | 184 |
| **Name of primer** | **Sequence (5'→ 3')** | **Anticipated PCR product (bp)** |
| CaWRKY48-F | CAGGGATGACAAAGAGGA |  |
| CaWRKY48-R | TATTCACCAACCAAGGGA | 167 |
| CaWRKY49-F | TAACCAATGCTCGCCAAATA |  |
| CaWRKY49-R | TGGTACGAAACTTTGGTGGA | 230 |
| CaWRKY50-F | AAATTTGATCAAGGATGCCA |  |
| CaWRKY50-R | TTTCCACCGTCATATCCAAA | 223 |
| CaWRKY51-F | CTGGGAGCACAGAGGAGGT |  |
| CaWRKY51-R | CAAGTTGCGCTTTGGAGG | 296 |
| CaWRKY52-F | CAGGGAGCACAGAGGAGGT |  |
| CaWRKY52-R | GAGGCTTTGGGTGGTTGT | 240 |
| CaWRKY53-F | ATGGCGGAGAATGAAGGA |  |
| CaWRKY53-R | CGTAGATGGTGGCGAGAA | 267 |
| CaWRKY54-F | ATTATCACCTTTACCCTCAC |  |
| CaWRKY54-R | AGCAATCCCTCATCAGTT | 180 |
| CaWRKY55-F | TGCTAACAACTTTGGGTGAAA |  |
| CaWRKY55-R | CTGGTTGTGATGATTTCCCTT | 205 |
| CaWRKY56-F | CCTAACCCTAATAATGGA |  |
| CaWRKY56-R | ATGAGTAGTTGTTGGAGC | 195 |
| CaWRKY57-F | ATGAATATCCCTCCCTCT |  |
| CaWRKY57-R | TCTTGATTGATGAAACCC | 226 |
| CaWRKY58-F | TTACCCACCACTATTTCC |  |
| CaWRKY58-R | ATTTCATCACATCGTCCT | 188 |
| CaWRKY59-F | CCACCACTATTTCCATCA |  |
| CaWRKY59-R | TTCATCACATCGTCCTCA | 182 |
| CaWRKY60-F | TGGCTTTAGATTTGTTTGCG |  |
| CaWRKY60-R | AGGCCGGTTAAGCATAGAGA | 170 |
| CaWRKY61-F | GATGGATACAAATGGAGGAA |  |
| CaWRKY61-R | ATGGCAATAAATGACAAAGG | 195 |
