# Supplementary material for: Comparative Genomic Analysis Reveals Extensive Genetic Variations of WRKYs in Solanaceae and Functional Variations of CaWRKYs in Pepper
Source: Front Genet. 2019 May 28;10:492. doi: 10.3389/fgene.2019.00492 (PMC6546733; doi:10.3389/fgene.2019.00492)
Supplement: TABLE S5 — Primers of CaWRKY genes used for VIGS analysis. [file Table_5.DOCX]

Supplemental Table S5 Primers of *CaWRKY* genes used for VIGS analysis

| **Name of primer** | **Sequence (5'→ 3')** |  | **Anticipated PCR product (bp)** |
| --- | --- | --- | --- |
| CaWRKY22-F | CGCGGATCCATGGAGGAGATTGAGGAA | BamHI |  |
| CaWRKY22-R | CCGGAATTCGTTGGCTAAAGTAAGAGTGC | EcoRI | 333 |
| CaWRKY45-F | CCGCTCGAGCAACAGGAACTGGGGATT | XhoI |  |
| CaWRKY45-R | CGCGGATCCTTGGAGCAGAAGAGTGAAAT | BamHI | 335 |
| CaWRKY58-F | CGCGGATCCACTCATGCAAAAGAGGTT | BamHI |  |
| CaWRKY58-R | CCGGAATTCTCAAAGTTATCGGTAGACT | EcoRI | 296 |
